# Orthology and near-cographs in the context of phylogenetic networks

**DOI:** 10.1186/s13015-025-00285-7

**Published:** 2025-10-02

**Authors:** Anna Lindeberg, Guillaume E. Scholz, Nicolas Wieseke, Marc Hellmuth

**Affiliations:** 1 Department of Mathematics, Stockholm University, Albanovägen 28, 10691 Stockholm, Sweden; 2 Department of Computer Science & Interdisciplinary Center for Bioinformatics, Universität Leipzig, Härtelstraße 16-18, 04107 Leipzig, Germany; 3 Department of Computer Science, Universität Leipzig, Augustusplatz 10, 04109 Leipzig, Germany

**Keywords:** Modular decomposition, Near cograph, Homology, Twin-width, Perfect graph, Linear-time algorithm, Level-1 network

## Abstract

Orthologous genes, which arise through speciation, play a key role in comparative genomics and functional inference. In particular, graph-based methods allow for the inference of orthology estimates without prior knowledge of the underlying gene or species trees. This results in orthology graphs, where each vertex represents a gene, and an edge exists between two vertices if the corresponding genes are estimated to be orthologs. Orthology graphs inferred under a tree-like evolutionary model must be cographs. However, real-world data often deviate from this property, either due to noise in the data, errors in inference methods or, simply, because evolution follows a network-like rather than a tree-like process. The latter, in particular, raises the question of whether and how orthology graphs can be derived from or, equivalently, are *explained by* phylogenetic networks. In this work, we study the constraints imposed on orthology graphs when the underlying evolutionary history follows a phylogenetic network instead of a tree. We show that any orthology graph can be represented by a sufficiently complex level-k network. However, such networks lack biologically meaningful constraints. In contrast, level-1 networks provide a simpler explanation, and we establish characterizations for level-1 explainable orthology graphs, i.e., those derived from level-1 evolutionary histories. To this end, we employ modular decomposition, a classical technique for studying graph structures. Specifically, an arbitrary graph is level-1 explainable if and only if each primitive subgraph is a near-cograph (a graph in which the removal of a single vertex results in a cograph). Additionally, we present a linear-time algorithm to recognize level-1 explainable orthology graphs and to construct a level-1 network that explains them, if such a network exists. Finally, we demonstrate the close relationship of level-1 explainable orthology graphs to the substitution operation, weakly chordal and perfect graphs, as well as graphs with twin-width at most 2.

## Introduction

In phylogenetics, the concept of homology describes genes that share a common ancestry, meaning they diverged from a common ancestral gene in an ancestral species. Homology between two genes *x* and *y* can be further classified based on the type of evolutionary event that caused the divergence of their most recent common ancestor. If the divergence occurred due to speciation where the ancestral species split into two descendant species, genes *x* and *y* are *orthologs* [1]. Homologous genes can also arise through evolutionary events other than speciation, such as gene duplication or horizontal gene transfer. In these cases, the genes are referred to as paralogs and xenologs, respectively [2, 3].

Orthologs are special in that they do not merely represent gene copies found in different species; they often share higher similarities in sequence or structure compared to paralogs or xenologs [4] and are generally assumed to perform more similar functions in their respective organisms [5]. As a result, orthology data is a valuable resource in areas such as functional prediction [6], genetic diagnostics [7], and even drug design [8], enabling researchers to transfer knowledge gained from genes in one species to their orthologous counterparts in another. Since the first definition of orthology in the early 1970 s [1], inferring orthologous relationships from genomic data has been an active field of research. Two main approaches have emerged to address this task: tree-based reconciliation methods and graph-based inference methods (see [9–11] for overviews).

Tree-based methods typically require input gene and species phylogenies (trees or networks) and reconcile these two phylogenies by computing a mapping from the gene phylogeny onto the species phylogeny. While maximum likelihood [12] or Bayesian approaches [13] exist, most methods employ event-based maximum parsimony, see [14] for an overview. This involves assigning costs to certain evolutionary events (e.g., duplication, speciation, and transfer) and finding a reconciliation that minimizes the total cost of all events. However, finding a cost-optimal reconciliation is computationally challenging [15], making it impractical for genome wide data sets containing thousands of genes. Additionally, tree-based methods are often sensitive to the input phylogenies and the cost scheme used. Once a phylogenetic reconciliation is computed, the orthology relationships can be directly inferred from the mapping.

**Fig. 1:**
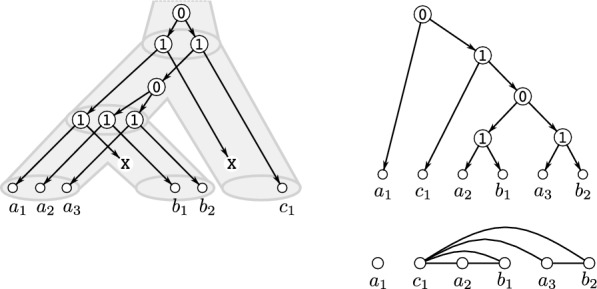
An evolutionary scenario, i.e., a species tree (drawn as tube-like tree) together with a reconciled gene tree is shown on the left. The observable part of the underlying gene tree is shown in the upper right part. Speciation and duplication vertices are marked with “1” and “0”, respectively. The orthology graph comprising all pairs of orthologous genes is drawn below the gene tree

Graph-based methods, in contrast, are used to infer an *orthology graph*, where each vertex corresponds to a gene, and an edge exists between two vertices if the corresponding genes are estimated to be orthologs, see Fig. 1 for an example. These methods aim to construct the orthology graph directly from pairwise sequences comparisons, bypassing the need for explicit gene and species phylogenies. Many of the commonly used methods for orthology-identification, such as OrthoMCL [16], ProteinOrtho [17, 18], OMA [19], or eggNOG [20], belong to this class. Extensive benchmarking [21, 22] has shown that these tools perform at least as well as tree-based methods such as Orthostrapper [23], PHOG [24], EnsemblCompara [25], or HOGENOM [26]. In particular, graph-based methods are are often more computationally efficient [11]. Early graph-based methods inferred orthology by identifying best matches or bi-directional best hits to compute clusters of orthologs [27, 28]. While effective as a starting point, these approaches do not account for whether the underlying evolutionary history is tree-like or, more generally, network-like.

This raises the question of whether such inferred orthology graphs are biologically feasible, namely whether there exist evolutionary scenarios that support these estimations. In the simplest setting, we may ask whether there exists a gene tree with internal nodes labeled as 1 (speciation) or 0 (non-speciation) such that two genes, *x* and *y*, are linked by an edge in the orthology graph if and only if their least common ancestor is labeled with 1. In this scenario, the orthology graph is biologically feasible if and only if it is a so-called cograph, i.e., a graph that does not contain an induced path on four vertices [29, 30]. In this case, the corresponding cotree represents a partially resolved version of the underlying gene tree. However, inferred orthology graphs often fail to satisfy the cograph property. One reason for this may be noise in the data or limitations in the inference methods. Assuming a phylogenetic tree-like model of evolution, we can leverage the structural property of true orthology graphs being cographs to correct errors in the inferred orthology graph, for example, by editing it to the closest cograph [30, 31]. Another reason could be that the evolutionary history is not tree-like. Although phylogenetic trees remain the most widely used evolutionary model, they are a simplification. It is well-acknowledged that genes and species may evolve in a network-like manner due to events such as hybridization (at the species level) or gene fusion (at the genetic level) [32, 33]. Various classes of phylogenetic networks have been studied, including galled trees, level-*k* networks, tree-child networks and many more [34–36].

Thus, it is natural to ask what constraints a particular phylogenetic network model imposes on the orthology graph. This question was addressed for trees [29] and so-called galled trees in [37–40] in which each vertex belongs to at most one “undirected cycle”. In this work, we extend the analysis to the broader class of level-*k* networks, with a particular focus on the prominent subclass of level-1 networks. We demonstrate that any orthology graph can be explained by a level-*k* network if *k* is sufficiently large. Consequently, level-*k* networks with arbitrary *k* do not impose specific constraints on orthology graphs. In contrast, level-1 networks do impose constraints, revealing a close structural relationship to cographs.

This paper is organized as follows. In “Preliminaries” section, we provide the basic definitions needed throughout this work. We then continue in “Graphs and orthologs explained by DAGs, networks and trees” section to explain in more detail how orthology graphs can be explained by DAGs or networks. We first review some of the results established to characterize those orthology graphs that can be explained by labeled phylogenetic trees, which are cographs. Assuming that an orthology graph is not a cograph, it cannot be explained by a tree; however, there is always a set of vertices that, if removed from *G*, result in a cograph. We use this result to provide a novel construction for networks explaining orthology graphs. However, these networks suffer from high complexity, in particular, they contain a high number of hybrid vertices, representing reticulate events. In “Level-1 explainable graphs” section, we focus on orthology graphs that can be explained by networks with relatively few hybrid vertices per “block”, specifically level-1 networks. After a brief overview of modular decomposition, a prominent and highly useful graph-theoretical tool in this context, we first examine so-called primitive graphs. A primitive graph *G* has only a “trivial” modular decomposition and, in particular, cannot be written as a join or a disjoint union of any two graphs. In this sense, primitive graphs differ significantly from cographs, which are precisely the graphs that can be constructed through a series of joins and disjoint unions. Nevertheless, understanding primitive graphs that can be explained by level-1 networks provides the foundation needed to characterize general level-1 explainable graphs. As Theorem 4.12 demonstrates, a primitive graph can be explained by a level-1 network if and only if it is a primitive near-cograph, meaning that there exists a vertex whose removal results in a cograph. This result enables us to establish multiple characterizations of general orthology graphs that can be explained by level-1 networks. More precisely, we prove that an orthology graph *G* is level-1 explainable if and only if

... every induced subgraph of *G* is level-1 explainable (Proposition 4.14)
... for all non-trivial so-called prime modules in the modular decomposition of *G*, a well-defined quotient graph is a near-cograph (Theorem 4.19)
... every primitive induced subgraph of *G* is a near-cograph (Theorem 4.20)
... *G* is explained by a *phylogenetic* level-1 network (Theorem 4.21)
... *G* can be constructed from a finite sequence of disjoint unions, joins and vertex substitutions (Theorem 4.24 & Theorem 4.26).

Furthermore, we present a linear-time algorithm for recognizing level-1 explainable orthology graphs and to construct a level-1 network that explains them, if such a network exists (Theorem 4.22). Finally, we show that the class of level-1 explainable graphs is a subclass of the class of graphs with twin-width at most two, as well as the class of weakly chordal graphs, and therefore, the class of perfect graphs. Since both perfect graphs and graphs of bounded twin-width possess interesting algorithmic properties [41–43], these results suggest that graphs explained by level-1 networks may also be of interest beyond the scope of orthology graphs. We discuss this and other potential directions for future research in the concluding “Summary and outlook” section.

## Preliminaries

### Sets

All sets considered in this paper are assumed to be finite. We denote with $$\left( {\begin{array}{c}X\\ 2\end{array}}\right) $$ the two-element subsets of a non-empty set *X* and with $2^X$ the powerset of *X*. A subset $\mathfrak {S}\subseteq 2^X$ is also called a *set system *(*on X)*. For a given set system $\mathfrak {C}$ on *X* and a non-empty subset $W\subseteq X$, we define

$$\begin{aligned} \mathfrak {C} \cap W {:}{=} \{C\cap W :C\in \mathfrak {C} \text { and } C \cap W\ne \emptyset \}. \end{aligned}$$

In particular, for $x\in X$ we put $\mathfrak {C}-x {:}{=} \mathfrak {C}\cap (X\setminus \{x\}) =\{C\setminus \{x\} :C\in \mathfrak {C} \text { and } C\ne \{x\}\}$.

A set system $\mathfrak {S}$ on *X* is *grounded* if $\emptyset \notin \mathfrak {S}$ and $\{x\}\in \mathfrak {S}$ for each $x\in X$, and $\mathfrak {S}$ is a *clustering system* if it is grounded and $X\in \mathfrak {S}$. Two sets *X* and *Y* *overlap* if $X\cap Y\notin \{X,Y,\emptyset \}$. A clustering system that contains no two elements that overlap is a *hierarchy*.

Let $\mathfrak {C}$ be a clustering system on *X*. Then, $\mathfrak {C}$ on *X* is *closed* if, for all *non-empty* $A\in 2^X$, it holds that $$\bigcap _{\begin{array}{c} C\in \mathfrak {C},\, A \subseteq C \end{array}} C =A \iff A\in \mathfrak {C}$$. A well known-characterization of closed clustering system is as follows, cf. e.g. [36, L. 16]

#### Lemma 2.1

A clustering system $\mathfrak {C}$ is closed if and only if $A,B \in \mathfrak {C}$ and $A\cap B\ne \emptyset$ implies $A\cap B\in \mathfrak {C}$.

Furthermore, $\mathfrak {C}$ satisfies property (L) if $C_1\cap C_2=C_1\cap C_3$ for all $C_1,C_2,C_3\in \mathfrak {C}$ where $C_1$ overlaps both $C_2$ and $C_3$. $\mathfrak {C}$ is *pre-binary* if, for all $x,y\in X$, there is a unique inclusion-minimal element $C\in \mathfrak {C}$ such that $x,y \in C$. Moreover, $\mathfrak {C}$ is *binary* if it is pre-binary and, for all $C\in \mathfrak {C}$, there are $x,y\in X$ such that *C* is the unique inclusion-minimal element in $\mathfrak {C}$ with $x,y\in C$.

### Graphs, di-graphs, DAGs and networks

An *(undirected) graph* $G=(V,E)$ consists of a non-empty *vertex set* $V(G){:}{=} V$ and an *edge set* $$E(G){:}{=} E \subseteq \left( {\begin{array}{c}V\\ 2\end{array}}\right) $$.

#### Remark

We emphasize that every undirected graph *G* can be interpreted as an orthology graph, that is, *V*(*G*) represents a set of genes, and $\{x, y\} \in E(G)$ if and only if two distinct genes $x, y \in V(G)$ are estimated to be orthologs. Therefore, instead of using the term “orthology graph”, we will simply use the term “graph”.

A *subgraph* *H* of *G* is a graph (*W*, *F*) that satisfies $W\subseteq V$ and $F\subseteq E$. A subgraph *H* of *G* is *induced* by $W\subseteq V$, denoted by $H =G[W]$, if $\{u,v\}\in E$ implies $\{u,v\}\in E(H)$ for all $u,v\in V(H)=W$. For a proper subset $W\subsetneq V(G)$, we denote with $G-W=G[V(G)\setminus W]$ the subgraph of *G* induced by $V(G)\setminus W$ and use the simplified notation $G-v{:}{=} G-\{v\}$. A *path* or $v_1v_k$*-path* in *G* is a is a sequence $v_1,\dots ,v_k$ of pairwise distinct vertices satisfying $\{v_i,v_{i+1}\}\in E(G)$ for $i\in \{1,\dots ,k-1\}$. A path on four vertices is called a $P_4$. A graph is *connected* if, for all vertices *u*, *v* in *G*, there is a *uv*-path.

The *complement *${\overline{G}}$ of a graph $G =(V,E)$ is the graph ${\overline{G}} {:}{=} (V, \{\{x,y\}:x,y \in V, x\ne y, \{x,y\}\notin E \} )$. The *join* of two vertex-disjoint graphs $H=(V,E)$ and $H'=(V',E')$ is defined by , whereas their *disjoint union* is given by .

A *directed graph* $N=(V,E)$ consists of a non-empty vertex set $V(N){:}{=} V$ and an edge set $E(N){:}{=} E\subseteq \{(u,v)\mid u,v\in V \text { and } u\ne v\}$. A *directed path* in a directed graph *N* is a sequence $v_1,\dots ,v_k$ of pairwise distinct vertices satisfying $(v_i,v_{i+1})\in E(N)$ for $i\in \{1,\dots ,k-1\}$. We also call such a directed path a *directed *$v_1v_n$*-path*. A *directed acyclic graph (DAG)* *N* is a directed graph that does not contain directed cycles, that is, there is no directed *uv*-path such that $(v,u)\in E(N)$. A directed graph *N* is *connected* if its underlying undirected graph $N^U$ is connected, where $N^U = (V(N), E(N^U))$ is obtained from *N* by replacing each edge $(u,v)\in E(N)$ by the edge $\{u,v\}$.

Two undirected (resp., directed) graphs *G* and *H* are *isomorphic*, in symbols $G\simeq H$, if there is a bijection $\varphi :V(G)\rightarrow V(H)$ such that for all $u,v\in V(G)$ it holds that $\{u,v\}\in E(G)$ if and only if $\{\varphi (u),\varphi (v)\}\in E(H)$ (resp., $(u,v)\in E(G)$ if and only if $(\varphi (u),\varphi (v))\in E(H)$).

We can associate a partial order $\preceq _N$ on the vertex set *V*(*N*) with a DAG *N*, defined by $v\preceq _N w$ if and only if there is a directed *wv*-path in *N*. In this case, we say that *w* is an *ancestor* of *v* and *v* is a *descendant* of *w*. If $v\preceq _N w$ and $v\ne w$, we write $v\prec _N w$. If $(u,v) \in E(N)$, then *u* is a *parent* of *v* and *v* a *child* of *u*. The sets ${{\,\textrm{parent}\,}}_N(v)$ and ${{\,\textrm{child}\,}}_N(v)$ comprise all parents and children of *v*, respectively. Two vertices $u,v\in V(N)$ are $\preceq _N$*-comparable* if $u\preceq _N v$ or $v\preceq _N u$ and, otherwise, *u* and *v* are $\preceq _{N}$*-incomparable*.

Let $N=(V,E)$ be a DAG. A vertex with $v\in V$ is a *leaf* if ${{\,\textrm{outdeg}\,}}_N(v)=0$, a *root* if ${{\,\textrm{indeg}\,}}_N(v)=0$, a *hybrid vertex* if ${{\,\textrm{indeg}\,}}_N(v)>1$, and *tree vertex* if ${{\,\textrm{indeg}\,}}_N(v)\le 1$. Equivalently, a leaf of *N* is a $\preceq _N$-minimal vertex, while a root of *N* is a $\preceq _N$-maximal vertex. The set of leaves is denoted by *L*(*N*). Moreover, the vertices that are not leaves are called *inner vertices*, and comprised in the set $V^0(N){:}{=} V(N)\setminus L(N)$. A DAG *N* *on X* is a DAG with leaf-set $L(N)=X$. Note that leaves and inner vertices can be hybrid vertices or tree vertices.

Following [36], we define (phylogenetic) networks here as a slightly more general class of DAGs than what is customarily considered in most of the literature on the topic.

#### Definition 2.2

A *network* is a DAG $N=(V,E)$ such that

1. There is a unique root of *N*, denoted by $\rho _N$.

A network *N* is *phylogenetic* if

(N2) There is no vertex $v\in V$ such that ${{\,\textrm{outdeg}\,}}_N(v)=1$ and ${{\,\textrm{indeg}\,}}_N(v)\le 1$.

For every vertex $v\in V(N)$ in a DAG *N*, the set of its descendant leaves $\texttt{C}_N(v){:}{=}\{ x\in L(N)\mid x \preceq _N v\}$ is a *cluster* of *N*. We write $\mathfrak {C}_N{:}{=}\{\texttt{C}_N(v)\mid v\in V(N)\}$ for the set of all clusters in *N*. Note that $\mathfrak {C}_N$ is a grounded set system on *L*(*N*) for every DAG *N* [44]. Moreover, $\mathfrak {C}_N$ is a clustering system for every network *N*; cf. [36, Lemma 14].

For a given a DAG *N* and a non-empty subset $A\subseteq L(N)$, a vertex $v\in V(N)$ is a *common ancestor of A* if *v* is ancestor of every vertex in *A*. Moreover, *v* is a *least common ancestor* (LCA) of *A* if *v* is a $\preceq _N$-minimal common ancestor of *A*. The set $\operatorname {LCA}_N(A)$ comprises all LCAs of *A* in *N*. We will, in particular, be interested in situations where $|\operatorname {LCA}_N(A)|=1$ holds for certain subsets $A\subseteq X$. For simplicity, we will write $\operatorname {lca}_N(A)=v$ in case that $\operatorname {LCA}_N(A)=\{v\}$ and say that $\operatorname {lca}_N(A)$* is well-defined*; otherwise, we leave $\operatorname {lca}_N(A)$ *undefined*. Moreover, we write $\operatorname {lca}_N(x,y)$ instead of $\operatorname {lca}_N(\{x,y\})$.

#### Lemma 2.3

( [45, L. 1 & Obs. 2]) Let *N* be a network on *X*. If $u,v\in V(N)$ satisfy $u\preceq _N v$, then $\texttt{C}_N(u)\subseteq \texttt{C}_N(v)$. Moreover, if $\operatorname {lca}_N(A)$ is well-defined for some non-empty subset $A\subseteq X$, then $\operatorname {lca}_N(A) \preceq _N v$ for all *v* with $A\subseteq \texttt{C}_N (v)$ and $\texttt{C}_N ( \operatorname {lca}_N (A))$ is the unique inclusion-minimal cluster in $\mathfrak {C}_N$ containing *A*.

The previous lemma highlights the connection between clusters in a network and the least common ancestor map. We now define specific properties of DAGs that rely on the LCAs and that play a central role in this contribution.

#### Definition 2.4

Let *N* be a DAG on *X*. Then, *N* has the *k-lca property* if $\operatorname {lca}_N(A)$ is well-defined for all nonempty subsets $A\subseteq X$ of size $|A|\le k$. A vertex $v\in V(N)$ is a *2-lca vertex* if $v = \operatorname {lca}_N(A)$ for some subset $A\subseteq X$ of size $|A|\le 2$. Moreover, *N* is *2-lca-relevant* if each vertex $v\in V(N)$ is a 2-lca vertex.

If *N* is a network with |*X*|-lca property, then it is simply called *lca-network*.

### Blocks and level-*k* networks

A directed graph is *biconnected* if it contains no vertex whose removal disconnects the graph. Note that biconnected directed graphs are necessarily connected. A *block* in a directed graph is an (inclusion) maximal biconnected subgraph. A block *B* is called *non-trivial* if it is not a single vertex or a single edge.

Note that a non-trivial block *B* in a DAG *N* may have several $\preceq _N$-maximal elements. This situation changes in networks. We summarize here some useful results for later reference.

#### Lemma 2.5

( [36, L. 8, 9 & 18, Obs. 1]) Every block *B* in a network *N* has a unique $\preceq _N$-maximal vertex $\max _B$. In particular, for every $w \in V(B)$, there is a directed path from $\max _B$ to *w* and every such path is completely contained in *B*.

 If *u* and *v* are distinct vertices of a block *B* and a block $B'$, then $B=B'$. If *v* is contained in block *B* and $B'$ but $v\notin \{\max _B, \max _{B'}\}$, then $B=B'$.

 If *u* and *v* are $\preceq _N$-incomparable vertices in *N* such that $\texttt{C}_N(v)\cap \texttt{C}_N(u)\ne \emptyset$, then *u* and *v* are contained in a common non-trivial block *B* in *N*. In this case, there is a hybrid vertex *h* in *N* that satisfies $h\prec _N u$ and $h\prec _N v$

#### Definition 2.6

A network *N* is *level-k* if each block *B* of *N* contains at most *k* hybrid vertices distinct from $\max _B$.

A level-0 network is a *tree*. In what follows, we are mainly interested in trees and level-1 networks. Lemma 50 together with Corollary 21 in [36] imply

#### Lemma 2.7

For every level-1 network *N* the clustering system $\mathfrak {C}_N$ is binary.

#### Lemma 2.8

( [36, L. 49, Thm. 8]) If *N* is a level-1 network, then $\mathfrak {C}_N$ is closed and *N* is an lca-network.

Lemma 2.8 implies, in particular, that every level-1 network has the 2-lca property.

#### Lemma 2.9

Let *N* be a phylogenetic level-1 network and $u,v\in V(N)$. Then, one of the following statements is true:

(i) $$\texttt{C}_N(v) = \texttt{C}_N(u)$$, $$\texttt{C}_N(u)\subset \texttt{C}_N(v)$$ or $$\texttt{C}_N(v)\subset \texttt{C}_N(u)$$, or
(ii) $\texttt{C}_N(u)\cap \texttt{C}_N(v) = \emptyset$ or $\texttt{C}_N(u)\cap \texttt{C}_N(v) = \texttt{C}_N(h)$ for some hybrid vertex $h \in V(N)$

#### Proof

If *u* and *v* are $\preceq _N$-comparable, then Lemma 2.3 implies that Condition (i) holds. Suppose now that *u* and *v* are $\preceq _N$-incomparable. By [36, Prop. 9], every phylogenetic level-1 network is a so-called tree-child network. This and [36, L. 34] implies that either $\texttt{C}_N(u)\cap \texttt{C}_N(v) = \texttt{C}_N(h)$ for some hybrid vertex $h \in V(N)$ or $\texttt{C}_N(u)\cap \texttt{C}_N(v) \notin \mathfrak {C}_N$. By Lemma 2.8, $\mathfrak {C}_N$ is closed for every level-1 network *N* and Lemma 2.1 implies that, in case that $\texttt{C}_N(u)\cap \texttt{C}_N(v) \notin \mathfrak {C}_N$, $\texttt{C}_N(u)\cap \texttt{C}_N(v) = \emptyset$ must hold. Hence, Condition (ii) holds. $\square$

### Hasse diagram and regular DAGs

If $\mathfrak {C}$ is a set system on *X*, then the *Hasse diagram* $\mathscr {H}(\mathfrak {C})$ (also known as the *cover digraph* of $\mathfrak {C}$ [46]) is the DAG with vertex set $\mathfrak {C}$ and directed edges (*A*, *B*) for each pair of vertices $A,B\in \mathfrak {C}$ such that (i) $B\subsetneq A$ and (ii) there is no $C\in \mathfrak {C}$ such that $B\subsetneq C\subsetneq A$. A DAG $N=(V,E)$ is *regular* if the map $\varphi :V\rightarrow V(\mathscr {H}(\mathfrak {C}_N))$ defined by $v\mapsto \texttt{C}_N(v)$ is an isomorphism of *N* and $\mathscr {H}(\mathfrak {C}_N)$ [47]. We summarize now some the structural properties of regular DAGs and networks.

#### Lemma 2.10

If *N* is a regular DAG, then *N* is phylogenetic and satisfies $\texttt{C}_N(v)\ne \texttt{C}_N(u)$ for all distinct $u,v\in V(N)$.

#### Proof

Let *N* be a regular DAG. Theorem 4.6 of [44] states that *N* is phylogenetic (c.f. [36, Thm. 2] for networks). Since $\varphi :V(N)\rightarrow \mathfrak {C}_N$ is an isomorphism between *N* and $\mathscr {H}(\mathfrak {C}_N)$, the map $\varphi$ is, in particular, injective. Thus, $u\ne v$ implies $\texttt{C}_N(u)=\varphi (u)\ne \varphi (v)=\texttt{C}_N(v)$ for all $u,v\in V(N)$. $\square$

One non-appealing property of the Hasse diagram $\mathscr {H}(\mathfrak {C})$ of a grounded set system $\mathfrak {C}$ on *X* is the fact that the leaves of $\mathscr {H}(\mathfrak {C})$ consist of the inclusion-minimal elements of $\mathfrak {C}$, that is, of the singletons $\{x\}$ for each $x\in X$. Consequently, we have $\mathfrak {C}\ne \mathfrak {C}_{\mathscr {H}(\mathfrak {C})}$. For practical reasons, we thus write $H\doteq \mathscr {H}(\mathfrak {C})$ for the directed graph that is obtained from $\mathscr {H}(\mathfrak {C})$ by relabeling all vertices $\{x\}$ in $\mathscr {H}(\mathfrak {C})$ by *x*. Whenever $\mathfrak {C}$ is a grounded set system, $H\doteq \mathscr {H}(\mathfrak {C})$ thus satisfies $\mathfrak {C}_H = \mathfrak {C}$.

For later reference, we provide two interesting results showing the connection of clustering systems of trees and level-1 networks with respect to their their underlying Hasse diagrams.

#### Theorem 2.11

([46, Sec. 3.5] and [36, Cor. 9]) Let $\mathfrak {C}$ be a clustering system on *X*. Then, there is a phylogenetic tree *T* on *X* such that $\mathfrak {C}_T = \mathfrak {C}$ if and only if $\mathfrak {C}$ is a hierarchy. In this case, $H\doteq \mathscr {H}(\mathfrak {C})$ is a phylogenetic tree on *X*.

#### Theorem 2.12

([36, Prop. 18, Cor. 36]) Let $\mathfrak {C}$ be a clustering system on *X*. Then, there is a phylogenetic level-1 network *N* on *X* such that $\mathfrak {C}_N = \mathfrak {C}$ if and only if $\mathfrak {C}$ is a closed and satisfies (L). In this case, $H\doteq \mathscr {H}(\mathfrak {C})$ is a phylogenetic level-1 network on *X*.

## Graphs and orthologs explained by DAGs, networks and trees

We will consider *labeled* DAGs (*N*, *t*), that is, DAGs $N=(V,E)$ equipped with a (vertex-)*labeling* $t:V^0(N)\rightarrow \Upsilon$ where $\Upsilon$ denotes some set of labels. In case that $\Upsilon =\{0,1\}$ we call (*N*, *t*) also 0/1*-labeled* and *t* a 0/1*-labeling*. If *N* is a DAG on *X* that has the 2-lca property, then we can derive from any 0/1-labeled version (*N*, *t*) the graph

$\mathscr {G}(N,t) =(X,E)$, where $\{x,y\}\in E$ if and only if $t(\operatorname {lca}_N(x,y))=1$ and $x,y\in X$.

### Definition 3.1

Let (*N*, *t*) be 0/1-labeled DAG on *X* and assume that *N* has the 2-lca property. A graph $G = (X,E)$ is *explained by* (*N*, *t*) if $G = \mathscr {G}(N,t)$. For the trivial case $|X|=1$, we say that $G=(X,\emptyset )$ is explained by (*N*, *t*) where $N=(X,\emptyset )$ is the single vertex graph and *t* is arbitrary with empty domain.

If $G = \mathscr {G}(N,t)$ and *N* is a level-*k* network, then *G* is *level-k explainable*, in short, Lev-*k*-Ex.

**Fig. 2:**
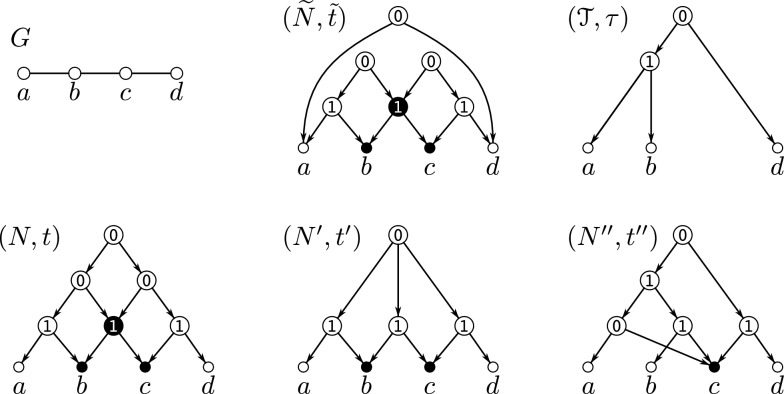
Shown are four 0/1-labeled DAGs (*N*, *t*), $(N',t')$, $(N'',t'')$ and $({\widetilde{N}}, {\tilde{t}})$ that all explain the graph *G*. Hybrid vertices within these DAGs are indicated as solid black vertices. Here, *N*, $N'$ and $N''$ are networks while ${\widetilde{N}}$ is not. Since $G\simeq P_4$, Theorem 3.3 implies that *G* cannot be explained by a 0/1-labeled tree. The network (*N*, *t*) is a “half-grid” (cf. [52]) and is level-3, whereas $(N',t')$ is a regular level-2 network with $N'\doteq \mathscr {H}(\mathfrak {C}_G)$ where $\mathfrak {C}_G$ is chosen according to Eq. (1). The network $(N'',t'')$ is a level-1 network and coincide with $(\mathscr {T}_{{\nwarrow }c},\tau _{{\nwarrow }c})$ that is obtained from the cotree $(\mathscr {T},\tau )$ (shown in the upper right corner) of the cograph $G-c$ (cf. Definition 3.7 and Theorem 3.9)

**Fig. 3:**
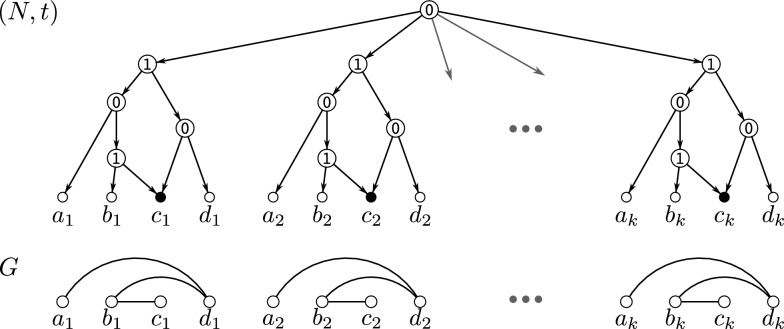
A 0/1-labeled level-1 network (*N*, *t*) where $G=\mathscr {G}(N,t)$ consists of $k>1$ vertex disjoint induced $P_4$s. If $G-Y$ is a cograph, then $Y\subseteq V(G)$ with $|Y|\ge k>1$ must hold. Hybrid vertices within *N* are indicated as solid black vertices

Examples illustrating Definition 3.1 are provided in Figs. 2 and 3. Clearly, if $G = \mathscr {G}(N,t)$, then its complement ${\overline{G}}$ is explained by the 0/1-labeled network $(N,{\overline{t}})$ for which ${\overline{t}}(v) = 1$ if and only if $t(v)=0$ for all $v\in V^0(G)$. For later reference, we summarize this into

### Observation 3.2

$G = \mathscr {G}(N,t)$ if and only if ${\overline{G}} = \mathscr {G}(N,{\overline{t}})$.

### Existing constructions

An important role in this contribution is played by graphs that can be explained by a 0/1-labeled tree, namely, cographs. The class of *cographs* is the smallest class of graphs that contains the single-vertex graph and is closed under the join- and disjoint union operators (also known as series and parallel composition) [48]. Cographs can be characterized as follows.

#### Theorem 3.3

([49–51]) Given a graph *G*, the following statements are equivalent:

(i) *G* is a cograph.
(ii) ${\overline{G}}$ is a cograph.
(iii) *G* does not contain an induced path $P_4$ on four vertices.
(iv) Every induced subgraph of *G* is a cograph.
(v) $G = \mathscr {G}(T,t)$ for some 0/1-labeled tree (*T*, *t*). In particular, there is a unique (up to isomorphism) 0/1-labeled phylogenetic tree in which no two adjacent vertices of the tree have the same label, called *cotree*, that explains *G*.

By Theorem 3.3, any graph that contains an induced $P_4$ cannot be explained by a 0/1-labeled tree. This naturally raises the question of which types of graphs can be explained by which types of 0/1-labeled networks. In [52], it was shown that every graph can be explained by a so-called 0/1-labeled half-grid (see the network (*N*, *t*) in Fig. 2 for an illustrative example). However, such half-grids are rather complex, as their level is $k \in \Omega (|X|^2)$.

A simpler construction, closely related to the one provided in the proof of [38, Prop. 3.3], is as follows. For a given graph $G = (X, E)$, define the set system

1 $$\begin{aligned} \begin{aligned} \mathfrak {C}_G {:}{=} \{X\} \cup \{&\{x, y\} :x, y \in X \text { and } \\  &(x = y \text { or } \{x, y\} \in E)\}. \end{aligned} \end{aligned}$$

By construction, $\mathfrak {C}_G$ is a clustering system. Now, consider the network $N \doteq \mathscr {H}(\mathfrak {C}_G)$ and a 2-element subset $\{x, y\}$ of *X*. If $\{x, y\} \in E$, then $\operatorname {lca}_N(x, y) = v$, where *v* is the unique vertex such that $\texttt{C}_N(v) = \{x, y\}$. If $\{x, y\} \notin E$, then $\operatorname {lca}_N(x, y) = \rho _N$. In particular, for every subset $Y \subseteq X$, $\operatorname {lca}_N(Y)$ is well-defined, i.e., *N* is an lca-network. Since *N* is regular, Lemma 2.10 implies that *N* is phylogenetic. Moreover, we can equip *N* with the 0/1-labeling *t* defined by $t(v) = 1$ for all $v \in V^0(N) \setminus \{\rho _N\}$ and $t(\rho _N) = 0$ to obtain the network (*N*, *t*) that explains *G* (see the network $(N',t')$ in Fig. 2 for an illustrative example). Furthermore, any hybrid in *N* must be a leaf, implying that *N* is level-*k* for $k \in O(|X|)$. The latter, in particular, implies

#### Proposition 3.4

For every graph $G = (X,E)$ there is a 0/1-labeled phylogenetic regular level-*k* lca-network (*N*, *t*) with $O(|X|+|E|)\subseteq O(|X|^2)$ vertices and $k\in O(|X|)$ that explains *G*.

Proposition 3.4 implies that every sparse graph $G = (X, E)$, i.e., a graph with $|E| \in O(|X|)$, can be explained by a network (*N*, *t*) having *O*(|*X*|) vertices. Moreover, for some dense graphs $G = (X, E)$, i.e., graphs with $|E| \in \Omega (|X|^2)$, their complement $\overline{G} = (X, \overline{E})$ is sparse. In this case, Proposition 3.4, together with Observation 3.2, implies that these types of graphs can also be explained by networks (*N*, *t*) having *O*(|*X*|) vertices. This property, however, is not guaranteed if both *G* and $\overline{G}$ are dense.

Proposition 3.4 implies, in particular, that the property of a graph being explainable by a 0/1-labeled lca-network is hereditary. As demonstrated later in Proposition 4.14, this hereditary property also applies to graphs explained by level-1 networks. The construction of a 0/1-labeled lca-network that explains induced subgraphs of a graph *G*, based on specific clusters in the network explaining *G*, is presented in the following result.

#### Proposition 3.5

( [38, Prop. 3.4]) Let (*N*, *t*) be a 0/1-labeled lca-network that explains *G* and $W\subseteq V(G)$ be a non-empty subset. Put $\mathfrak {C}'{:}{=} \mathfrak {C}_N\cap W$. Then, $\mathfrak {C}'$ is a clustering system on *W* and $N'\doteq \mathscr {H}(\mathfrak {C}')$ is an lca-network. Moreover, $(N',t')$ explains *G*[*W*], where $t'$ is the labeling defined by putting $t'(v){:}{=} t(\operatorname {lca}_N(\texttt{C}_N(v)))$ for all inner vertices *v* of *G*.

### Novel constructions

In what follows, we provide further methods to construct level-*k* networks, with $k < |X|$, that can explain a given graph $G = (X, E)$. To this end, we first consider subsets of clustering systems, denoted by $\mathfrak {C}(\{1,2\})$, associated with networks that explain a given graph, and their Hasse diagram $\mathscr {H}(\mathfrak {C}(\{1,2\}))$. More precisely, in [44, 53] DAGs that are 2-lca-relevant have been studied. These DAGs have strong connections to regular DAGs and in particular, can be efficiently derived from any DAG *G* by transforming *G* into a 2-lca-relevant one. For brevity, we omit the technical details and instead summarize the most important results from [44, 53] in the following theorem which, in addition, includes new results on networks explaining graphs.

#### Theorem 3.6

Let $G=(X,E)$ be a graph explained by a 0/1-labeled network (*N*, *t*) and define

$$\begin{aligned} \begin{aligned} \mathfrak {C}(\{1,2\}){:}{=} \{&C\in \mathfrak {C}_N\,:\, C \text { is the unique inclusion-} \\  &\text {minimal cluster in } \mathfrak {C}_N \text {containing } \\  &A \text { for some } A\subseteq X,\, |A|\in \{1,2\} \}. \end{aligned} \end{aligned}$$

Then, $\mathscr {H}(\mathfrak {C}(\{1,2\}))$ is a regular DAG that is 2-lca-relevant. In particular, there is a DAG $H \simeq \mathscr {H}(\mathfrak {C}(\{1,2\}))$ on *X* such that $V(H)\subseteq V(N)$ and $\operatorname {lca}_N(x,y)=\operatorname {lca}_{H}(x,y)$ for all $x,y\in X$. In addition, *H* can be equipped with a labeling $t'$ such that $(H,t')$ explains *G*. Moreover, if *N* is level-1, then *H* is a phylogenetic level-1 network.

#### Proof

Let $G=(X,E)$ be a graph explained by a 0/1-labeled network (*N*, *t*) and $\mathfrak {C}(\{1,2\})$ be defined as in the statement of the theorem. Note that *N* must have the 2-$\operatorname {lca}$ property. Thus, we can apply [53, Thm. 3.12] which states that $H \simeq \mathscr {H}(\mathfrak {C}(\{1,2\}))$ is a regular and 2-lca relevant DAG on *X* that satisfies $V(H)\subseteq V(N)$ and $\operatorname {lca}_N(x,y)=\operatorname {lca}_{H}(x,y)$ for all $x,y\in X$. Since $V(H)\subseteq V(N)$, we can define $t'{:}{=} V^0(H) \rightarrow \{0,1\}$ by putting $t'(v) {:}{=} t(v)$ for all $v\in V^0(H)$. Since $\operatorname {lca}_N(x,y)=\operatorname {lca}_{H}(x,y)$ for all $x,y\in X$, we can conclude that $t(\operatorname {lca}_{N}(x,y))=t'(\operatorname {lca}_H(x,y))$ for all $x,y\in X$. Hence, $\mathscr {G}(H,t')=G$ and it follows that $(H,t')$ explains *G*.

Finally assume that *N* is a level-1 network. By Lemma 2.7, $\mathfrak {C}_N$ is binary. Hence, $\mathfrak {C}(\{1,2\})=\mathfrak {C}_{N}$. Theorem 2.12 implies that $\mathfrak {C}(\{1,2\})$ is closed and satisfied (L) and that *H* is a phylogenetic level-1 network. $\square$

Theorem 3.6 and Proposition 3.5 are closely related, as both provide methods for constructing labeled DAGs and networks that explain a given graph *G* or a subgraph of *G*. Theorem 3.6 shows that any graph explained by a 0/1-labeled network can also be explained by a regular 2-lca-relevant DAG, with additional guarantees when the network is level-1. In contrast, Proposition 3.5 specifically requires the input network to be an lca-network and focuses on constructing a new lca-network that explains an induced subgraph *G*[*W*]. Thus, while both results address network constructions, Proposition 3.5 imposes a stronger assumption on the input network.

In the following, we present a simple method for extending a DAG *N* by introducing a new vertex *z* and applying a series of operations, resulting in the DAG $N_{{\nwarrow }z}$. This procedure is essential for extending a 0/1-labeled network to explain a larger graph while preserving its key structural properties. Definition 3.7 provides the formal details for constructing $N_{{\nwarrow }z}$. Next, Lemma 3.8 establishes important properties of $N_{{\nwarrow }z}$, including the preservation of network characteristics (e.g., phylogenetic or the 2-lca property) and showing that the level of $N_{{\nwarrow }z}$ increases by at most one compared to the number of hybrids of *N*. Finally, Theorem 3.9 demonstrates that for a graph $G - z$ explained by (*N*, *t*), the network $N_{{\nwarrow }z}$ can be equipped with a 0/1-labeling $t_{{\nwarrow }z}$, resulting in a labeled network $(N_{{\nwarrow }z}, t_{{\nwarrow }z})$ that explains the entire graph *G*.

#### Definition 3.7

Let *N* be a connected DAG on *X* and $v\in V(N)$. The *expanding operation *${{\,\mathrm{\textrm{EXPD}}\,}}(v)$ is defined as follows: create a new vertex $w_v$, replace edges (*u*, *v*) by $(u, w_v)$ for all $u \in {{\,\textrm{parent}\,}}_N (v)$, and add the edge $(w_v, v)$.

Let $z\notin V(N)$ be a vertex that is not part of *N*. If $|X|>1$, then the directed graph $N_{{\nwarrow }z}$ is obtained from *N* by

1. applying ${{\,\mathrm{\textrm{EXPD}}\,}}(x)$ on every $x\in X$ with ${{\,\textrm{indeg}\,}}_N(x)> 1$ and, afterwards,
2. replacing every edge (*u*, *x*) by the two edges $(u,u_x)$ and $(u_x,x)$ for all $x\in X$ and, afterwards,
3. adding the new vertex *z* together with edge $(u_x,z)$ for all $x\in X$.

If $|X|=1$, then $X =V(N)$, and we define $N_{{\nwarrow }z}$ as the network with a single root $u_x$ that has precisely the two children *z* and $x\in X$ and no other vertices or edges.

**Fig. 4:**
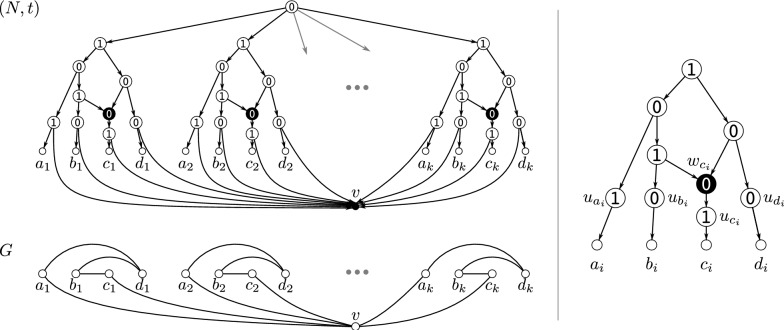
Example for Definition 3.7, constructing the shown 0/1-labeled network $(N_{{\nwarrow }v},t_{{\nwarrow }v})$ from the level-1 network (*N*, *t*) as shown in Fig. 3. The graph $G-v = \mathscr {G}(N,t)$ is explained by the level-1 network (*N*, *t*), however, the network $(N_{{\nwarrow }v},t_{{\nwarrow }v})$ is level-$(k+1)$, $k>1$. Note that *G* contains *k* induced cycles $C_5$ on five vertices and is, by Lemma 4.28, not Lev-1-Ex. Hybrid vertices within $N_{{\nwarrow }v}$ and *N* are indicated as solid black vertices

In the following, we always assume that for the constructed directed graph $N_{{\nwarrow }z}$ it holds that $z\notin V(N)$. Moreover, observe that $V(N) \subseteq V(N_{{\nwarrow }z})$ and $V(N_{{\nwarrow }z}){\setminus } V(N) = \{z\} \cup \{u_x:x\in X\} \cup \{w_x :x\in X \text { is a hybrid in } N \}$. Definition 3.7 is illustrated in Fig. 4.

#### Lemma 3.8

Suppose that *N* is a DAG on *X* with the 2-lca property. Then, $N_{{\nwarrow }z}$ is a DAG on $X\cup \{z\}$ with the 2-lca property that satisfies, for all $x,y\in X\cup \{z\}$,

2 $$\begin{aligned} \operatorname {lca}_{N_{{\nwarrow }z}}(x,y)= {\left\{ \begin{array}{ll} \operatorname {lca}_N(x,y)& \text {if } z\notin \{x,y\}\\ u_x &  \text {if } z=y\\ u_y &  \text {otherwise, i.e., } z=x. \end{array}\right. } \end{aligned}$$

Moreover, if *N* is a network, then $N_{{\nwarrow }z}$ is a network and, if *N* is phylogenetic, then $N_{{\nwarrow }z}$ is phylogenetic. In particular, if *N* is an $\operatorname {lca}$-network, then $N_{{\nwarrow }z}$ is an $\operatorname {lca}$-network. Furthermore, if *N* is a network that has at most $k-1$ hybrid vertices, then $N_{{\nwarrow }z}$ has at most *k* hybrids and is level-*k*.

#### Proof

Let *N* be a DAG on *X* with the 2-lca property. Clearly, *N* must be connected since if not, then at least two leaves have no common ancestor at all. It is straightforward to verify that the statements hold in case that $|X|=1$. Hence, suppose in the following that $|X|>1$. This and connectedness of *N* implies that $|E(N)|>1$. To simplify writing, put $N'{:}{=} N_{{\nwarrow }z}$ where $N_{{\nwarrow }z}$ is defined according to Definition 3.7. One easily observes that $N'$ is a DAG with leaf-set $X\cup \{z\}$. Note that by construction, $V(N)\subseteq V(N')$ and $w\preceq _N w'$ if and only if $w\preceq _{N'}w'$, for all $w,w'\in V(N)$. In particular, each new vertex $u\in V(N')\setminus V(N)$ satisfies $u=w_x$ or $u=u_x$ for some $x\in X$ or $u=z$ according to Step (1), (2) and (3) in Definition 3.7, respectively. Thus, $V(N')\setminus V(N) = \{z\} \cup \{u_x:x\in X\}\cup \{w_x:x\in X\text { and } {{\,\textrm{indeg}\,}}_N(x)>1\}$. By construction, $\texttt{C}_{N'}(u)=\{z,x\}$ for all $u\in V(N')\setminus (V(N) \cup \{z\})$ and $\texttt{C}_{N'}(z)=\{z\}$. Therefore, no vertex in $V(N')\setminus V(N)$ can be a common ancestor of two distinct elements in *X*.

Now let $x,y\in X$. Since *N* has the 2-lca property, $\operatorname {lca}_N(x,y)$ is well-defined. As previously argued, there is no common ancestor of *x* and *y* contained in $V(N')\setminus V(N)$, and since the partial order of *N* agrees with that of $N'$ on common ancestors of *x* and *y* in *N*, we can conclude that $\operatorname {lca}_N(x,y)=\operatorname {lca}_{N'}(x,y)$ for all $x,y\in X$. Next, consider *z* and some $x\in X$. There is, by construction of $N'$, a unique parent $u_x$ of *x* in $N'$. Moreover, $(u_x, z)$ is an edge of $N'$, hence $u_x$ is a common ancestor of *z* and *x* in $N'$. Since $u_x$ is the unique parent of *x* in $N'$, every other common ancestor *u* of *z* and *x* in $N'$ satisfy $u_x\prec _{N'} u$. Thus, $\operatorname {lca}_{N'}(x,z)$ is well-defined and satisfies $\operatorname {lca}_{N'}(x,z)=u_x$ for all $x\in X$. In summary, $N'$ has the 2-lca property.

Clearly, if *N* is a network, then the root $\rho$ of *N* remains the unique root in $N'$, i.e., $N'$ is a network. Now, assume that *N* is phylogenetic. By construction of $N'$, the only vertices that are in $V(N)\cap V(N')$ and that differ in their in- or out-degrees are those hybrids *x* in *N* that are leaves. Such vertices still have out-degree zero in $N'$. Moreover, *z* has ${{\,\textrm{indeg}\,}}_{N'}(z)>1$ since $|X|>1$. To recall, each new vertex $u\in V(N')\setminus (V(N)\cup \{z\})$ satisfies $u=u_x$ or $u=w_x$ for some $x\in X$. If $u=u_x$, then *u* has exactly two children *z* and *x* and thus, ${{\,\textrm{outdeg}\,}}_{N'}(u)>1$. If $u=w_x$ then ${{\,\textrm{indeg}\,}}_{N'}(w_x)={{\,\textrm{indeg}\,}}_N(x)>1$ is a direct consequence of the definition of the ${{\,\mathrm{\textrm{EXPD}}\,}}$-operation. Hence, in $N'$ there is no vertex *v* with ${{\,\textrm{indeg}\,}}(v)\le 1$ and ${{\,\textrm{outdeg}\,}}(v) = 1$, i.e., $N'$ remains phylogenetic.

Now suppose that *N* is an $\operatorname {lca}$-network, i.e., that $\operatorname {lca}_N(A)$ is well-defined for every non-empty subset $A\subseteq X$. We will now show that $N'$ is also an $\operatorname {lca}$-network. In particular, *N* has the 2-lca property and, as shown above, $N'$ has the 2-lca property. Using analogous arguments to those used to show that $\operatorname {lca}_{N'}(x,y)=\operatorname {lca}_N(x,y)$ for all $x,y\in X$ it is straightforward to verify that $\operatorname {lca}_{N'}(A)=\operatorname {lca}_N(A)$ for all non-empty subsets $A\subseteq X$. Now, consider the vertex *z* and some subset $A'\subseteq X\cup \{z\}$ that contains *z*. If $|A'|\in \{1,2\}$, then the fact that $N'$ has the 2-lca property implies that $\operatorname {lca}_{N'}(A')$ is well-defined. Hence, suppose that $|A'|\ge 3$. In this case, there is a subset $A\subseteq X\cap A'$ of size $|A|\ge 2$. Since *N* is an $\operatorname {lca}$-network, $\operatorname {lca}_N(A)$ is well-defined. In particular $A\subseteq X$ and, as previously argued, it holds that $\operatorname {lca}_{N'}(A)=\operatorname {lca}_{N}(A)$. Since $|A|>1$, it follows that $\operatorname {lca}_{N'}(A)=u$ for some $u\in V^0(N)$. By construction of $N'$, we have $z\prec _{N'}u_x\prec _{N'}u$ for every $x\in A$, so *u* is a common ancestor of $A'=A\cup \{z\}$. Moreover, since no vertex in $V(N')\setminus V(N)$ can be a common ancestor of *A* it follows that every common ancestor $u'$ of $A'$ must satisfy $u'\in V(N)$. Since $A \subseteq A'$, every such $u'$ is also a common ancestor of *A*, and $u = \operatorname {lca}_N(A)$ therefore implies that $u \preceq _N u'$. Since $u'$ was an arbitrary common ancestor of $A'$, the latter arguments imply that $\operatorname {lca}_{N'}(A') = \operatorname {lca}_N(A)$ and that $\operatorname {lca}_{N'}(A')$ is well-defined. In conclusion, $N'$ is an $\operatorname {lca}$-network.

Finally, assume that *N* has at most $k-1$ hybrid vertices. As argued above, the only vertices that are in $V(N)\cap V(N')$ and that differ in their in- or out-degrees are those hybrids *x* in *N* that are leaves and for each such hybrid *x* there is a unique corresponding hybrid vertex $w_x$ in $N'$ added during Step 3.7 of Definition 3.7 by applying ${{\,\mathrm{\textrm{EXPD}}\,}}(x)$. In particular, there is a 1-to-1 correspondence between hybrid-leaves *x* and the vertices $w_x$ resulting from ${{\,\mathrm{\textrm{EXPD}}\,}}(x)$. Each hybrid vertex in *N* that is not a leaf will, by construction, remain a hybrid vertex in $N'$. Since $|X|>1$, the added vertex *z* will serve as a hybrid in $N'$ that is not contained in *N*. The latter arguments imply that the total number of hybrids in $N'$ is at most *k*. This, in particular, implies that every block in $N'$ can have at most *k* hybrids. Consequently, $N'$ is level-*k*. $\square$

#### Theorem 3.9

Let *G* be a graph and suppose that (*N*, *t*) is a 0/1-labeled network that explains $G-z$ for some $z\in V(G)$. Then, $(N_{{\nwarrow }z}, t_{{\nwarrow }z})$ explains *G* where $t_{{\nwarrow }z} :V^0(N_{{\nwarrow }z}) \rightarrow \{0,1\}$ is the map defined by putting, for each $w\in V^0(N_{{\nwarrow }z})$,

3 $$\begin{aligned} t_{{\nwarrow }z}(w){:}{=}{\left\{ \begin{array}{ll} t(w) & \text {if } w\in V(N).\\ 1 & \text {if } w=u_x \text { for some } x\in X \text { and } \\   &  \{x,z\}\in E(G).\\ 0 & \text {otherwise, i.e., if either }w=w_x \\   & \text {or if }w=u_x\text { and }\{x,z\}\notin E(G)\\ & \text {for some } x\in X. \end{array}\right. } \end{aligned}$$

#### Proof

Let *G* be a graph and suppose that (*N*, *t*) is a 0/1-labeled network that explains $G-z$ for some $z\in V(G)$. Consider now $(N_{{\nwarrow }z},t_{{\nwarrow }z})$, where $t_{{\nwarrow }z}$ is defined as in Eq. (3). Let $x,y\in V(G)$. If $z\notin \{x,y\}$ then, according to Eq. (2) in Lemma 3.8, $\operatorname {lca}_{N_{{\nwarrow }z}}(x,y) = \operatorname {lca}_{N}(x,y){=}{:} w\in V(N)$ and thus, $t_{{\nwarrow }z}(w) = t(w)$. Since (*N*, *t*) explains $G-z$ and $z\notin \{x,y\}$ we can conclude that $t_{{\nwarrow }z}(w) =1$ if and only if $\{x,y\}\in E(G)$. Consider now a vertex $x\in V(G) \setminus \{z\}$ and the vertex *z*. By Eq. (2), $\operatorname {lca}_{N_{{\nwarrow }z}}(x,z) = u_x$. By definition of $t_{{\nwarrow }z}$, we have $t_{{\nwarrow }z}(\operatorname {lca}_{N_{{\nwarrow }z}}(x,z)) =1$ if and only if $\{x,y\}\in E(G)$. Consequently, $(N_{{\nwarrow }z},t_{{\nwarrow }z})$ explains *G*. $\square$

In phylogenetic trees, the number of inner vertices and edges is bounded from above (linearly) by the number of its leaves. In general, phylogenetic networks lack this property. In fact it is easy to construct networks on *X* with $2^{|X|}$ vertices or more. Nevertheless, as shown in [38, Prop. 3.3], it is always possible to construct a level-|*X*| network with $O(|X|^2)$ vertices that can explain a given graph $G=(X,E)$. We provide here a slightly stronger statement.

#### Theorem 3.10

If there is a subset $Y\subsetneq X$ of a graph $G=(X,E)$ such that $G-Y$ is a cograph, then *G* is Lev-| *Y* |-Ex and can be explained by a 0/1-labeled phylogenetic lca-network with $O((|Y|+1)|X|)\subseteq O(|X|^2)$ vertices. In particular, every graph $G=(X,E)$ is Lev-*k*-Ex for some $k<|X|$.

#### Proof

Let $G=(X,E)$ be a graph and assume that there is a set $Y\subsetneq X$ such that $G-Y$ is a cograph. If $|Y|=0$, then $G-Y=G$ is a cograph and, by Theorem 3.3, *G* is explained by a phylogenetic 0/1-labeled tree and thus, by a phylogenetic 0/1-labeled level-0 network with *O*(|*X*|) vertices. In particular, *G* is Lev-0-Ex. Moreover, any tree is an lca-network [36]. Consider now the case when $1\le |Y|<|X|$. Since $G-Y$ is a cograph, Theorem 3.3 implies that $G-Y$ is explained by its cotree and thus, re-using the previous arguments, by a phylogenetic level-0 lca-network $(N_0,t_0)$. Order the vertices $Y=\{y_1,\ldots ,y_k\}$ arbitrarily, where $k{:}{=} |Y|$. We can, by repeatedly applying Definition 3.7, define a sequence of *k* digraphs $N_1$, ..., $N_k$ by putting $N_i{:}{=} (N_{i-1})_{{\nwarrow }y_i}$ for $i=1,2\ldots , k$. By repeated application of Lemma 3.8 and Theorem 3.9 there is a labeling $t_i$ of the level-*i* network $N_i$ such that $\mathscr {G}(N_i,t_i)=G-\{y_{i+1},\ldots ,y_k\}$. In particular, $(N_k,t_k)$ is a phylogenetic level-*k* lca-network that explains $G-\emptyset =G$, that is, *G* is Lev-*k*-Ex. Moreover, when constructing $N_{i+1}$ from $N_i$ we add *O*(|*X*|) vertices. Hence, the final network $N_k$ has *O*(*k*|*X*|).

To prove the second statement, observe that for every graph $G=(X,E)$ and all sets $Y = X\setminus \{x\}$ for some $x\in X$ it holds that $G-Y$ is a single vertex graph and thus, a cograph. Reusing the arguments in preceding paragraph, *G* is Lev-*k*-Ex for some $k \le |X|-1$. $\square$

Finding a subset $Y \subsetneq V(G)$ of minimum size such that $G - Y$ is a cograph is an NP-hard task [54]. It remains, however, an open question whether the problem of finding the smallest *k* such that *G* is Lev-*k*-Ex is an NP-hard task as well. There is no direct relation between the size of a set *Y* for which $G - Y$ is a cograph and the *k* for which *G* is Lev-*k*-Ex. In particular, the converse of Theorem 3.10 is in general not satisfied, as illustrated in Fig. 3. In this example, *G* is Lev-1-Ex, however, the size of a minimum set *Y* resulting in a cograph $G - Y$ is $|Y| > 1$. A further example is provided in Fig. 6.

## Level-1 explainable graphs

In “Graphs and orthologs explained by DAGs, networks and trees” section, we saw that every graph can be explained by some 0/1-labeled network. Moreover, only cographs admit an explanation by a 0/1-labeled tree and, thus, by a level-0 network. Although the number of vertices in a network (*N*, *t*) explaining a graph $G = (X,E)$ can be asymptotically bounded above by a function of the number |*X*| of its leaves (cf. Theorem 3.10), such networks can still be quite complex. In particular, their level can increase drastically, leading to level-*k* networks, where *k* is close to |*X*|, as demonstrated by the construction in Theorem 3.9. This naturally raises the question of whether it is possible to find a “simpler” 0/1-labeled network that explains *G*. Hence, we explore in this section the structure of graphs that can be explained by level-1 networks.

To this end, we begin in “Modular decomposition” section by introducing the modular decomposition of graphs. Every graph admits a unique decomposition, $\mathbb {M}_{\textrm{str}}(G)$, into so-called strong modules. The set $\mathbb {M}_{\textrm{str}}(G)$ of these modules forms a hierarchy and can therefore be represented as a labeled tree, the modular decomposition tree $(\mathscr {T}_G,\tau _G)$. However, in general, $(\mathscr {T}_G,\tau _G)$ is not 0/1-labeled, as it uses an additional label “$\textrm{prime}$” for its vertices. In particular, *G* is a cograph if and only if $(\mathscr {T}_G,\tau _G)$ is 0/1-labeled. The main goal here is to determine whether a given graph *G* is Lev-1-Ex, and, if so, to provide a level-1 network that explains *G*. To achieve this, we propose replacing each $\textrm{prime}$-labeled vertex with a 0/1-labeled network. In “Characterizing general Lev-1-Ex graphs” section, we study the structure of so-called primitive graphs that are Lev-1-Ex. It turns out that these graphs are precisely the *near-cographs*, i.e., those graphs for which the removal of a single vertex results in a cograph. In “Characterizing primitive Lev-1-Ex graphs” section, we combine these findings to provide several characterizations of Lev-1-Ex graphs. In particular, this enables us to to design a linear-time algorithm for recognizing Lev-1-Ex graphs and constructing 0/1-labeled level-1 networks explaining them.

### Modular decomposition

We begin by providing the necessary definitions for characterizing graphs that are Lev-1-Ex. To recall, the class of cographs is precisely the class of graphs that can be explained by a 0/1-labeled tree. However, not all graphs are cographs. Once *G* contains an induced $P_4$, there is no 0/1-labeled tree (*T*, *t*) that can explain *G*. Nevertheless, there is a way to construct a labeled tree, called the *modular decomposition tree*, which captures at least partial information about the structure of *G*.

A *module* *M* of a graph $G=(X,E)$ is a non-empty subset $M\subseteq X$ such that for all $x,y\in M$ it holds that $N_G(x)\setminus M = N_G(y)\setminus M$, where $N_G(x)$ denotes the set of all vertices in *G* adjacent to *x*. In other words, *M* is a module if, for all $x,y\in M$ and all $z\notin M$, *x* is adjacent to *z* if and only if *y* is adjacent to *z*. Note that *X* and each singleton set $\{x\}$ for $x\in X$ are modules of *G*. They are called the *trivial* modules of *G*. All other modules are *non-trivial*. The set $\mathbb {M}(G)$ comprising all modules of *G* in particular contains the trivial modules of *G* and is therefore always non-empty. A graph *G* is called *primitive* if it has at least four vertices and $\mathbb {M}(G)$ contains trivial modules only. The smallest primitive graph is the path $P_4$ on four vertices. Moreover, every primitive graph contains an induced $P_4$ (c.f. [55, L. 1.5]) and is therefore not a cograph. Modules and modular decomposition have appeared under many different names, we refer to the surveys [56, 57] for recollections of the history of these concepts; in particular note that modules have also appeared under the names *closed sets* [58], *clumps* [59, 60], *autonomous sets* [61, 62] and *clans* [63–66]. Similarly, primitive graphs [63–65] are also known as *prime* graphs [56, 67] and *indecomposable* graphs [55, 68–70].

A module *M* of $G=(X,E)$ is *strong* if *M* does not overlap with any other module of *G*. Clearly, all trivial modules of *G* are strong. The set of strong modules $\mathbb {M}_{\textrm{str}}(G)\subseteq \mathbb {M}(G)$ is uniquely determined (see e.g. [66]) and $\mathbb {M}_{\textrm{str}}(G)$ is a hierarchy on *X*. One can furthermore distinguish between three types of strong modules; a module $M\in \mathbb {M}_{\textrm{str}}(G)$ is:

- *series* if $\overline{G[M]}$ is disconnected,
- *parallel* if *G*[*M*] is disconnected and
- *prime* otherwise, i.e., if both $\overline{G[M]}$ and *G*[*M*] are connected.

We can in particular study the strong modules of $G=(X,E)$ by considering the *modular decomposition tree* (MDT) of *G*, that is, the Hasse diagram $\mathscr {H}(\mathbb {M}_{\textrm{str}}(G))$. More precisely, we can equip $\mathscr {T}_G\doteq \mathscr {H}(\mathbb {M}_{\textrm{str}}(G))$ with a labeling $\tau _G:V^0(\mathscr {T}_G)\rightarrow \{0,1,\textrm{prime}\}$ defined by setting

$$\begin{aligned}\tau _G(M){:}{=} {\left\{ \begin{array}{ll} 0 &  \text {if }M\text { is a parallel module} \\ 1 &  \text {if }M\text { is a series module} \\ \textrm{prime}&  \text {if }M\text { is a prime module} \end{array}\right. } \end{aligned}$$

for all $M\in V^0(\mathscr {T}_G)=\mathbb {M}_{\textrm{str}}(G){\setminus }\{\{x\}\,:\,x\in X\}$. Since $\mathbb {M}_{\textrm{str}}(G)$ is a hierarchy, $\mathscr {T}_G$ is a phylogenetic tree (c.f. Theorem 2.11). Hence, $(\mathscr {T}_G, \tau _G)$ is a natural generalization of cotrees that, however, does in general not explain *G*. Although, for all distinct $x,y\in L(\mathscr {T}_G)=V(G)=X$ it holds that $\tau _G(\operatorname {lca}_{\mathscr {T}_G}(x,y))=1$ implies $\{x,y\}\in E(G)$ and $\tau _G(\operatorname {lca}_{\mathscr {T}_G}(x,y))=0$ implies that $\{x,y\}\notin E(G)$, the converse of these two implications is, in general, not satisfied. In particular, by Theorem 3.3 and the definition of cotrees, *G* is explained by $(\mathscr {T}_G, \tau _G)$ precisely if *G* is a cograph and thus, if $(\mathscr {T}_G, \tau _G)$ does not contain inner vertices *v* with $\tau _G(v)=\textrm{prime}$.

Since $\mathbb {M}_{\textrm{str}}(G)$ is uniquely determined for all graphs $G=(X,E)$ and since it does not contain overlapping modules, there is a unique partition $\mathbb {M}_{\max }(G) = \{M_1,\dots , M_k\}$ of *X* into $k\ge 2$ inclusion-maximal strong modules $M_j\ne X$ of *G*, whenever *G* contains $|X|\ge 2$ vertices [63, 64].

A well-known fact, that is easy to prove, is the following

#### Observation 4.1

Let *G* be a graph, $(\mathscr {T},\tau )$ be its MDT and *M* be a strong module of *G* such that $|M|>1$. Then $\mathbb {M}_{\max }(G[M]) = \{M_1,\dots , M_k\}$ contains $k>1$ elements. Moreover, If $\tau (M) = 0$, then  and if $\tau (M) = 1$, then  .

For strong modules *M* such that $\tau (M)=\textrm{prime}$, the situation is more complicated, but can be approached as follows. Two disjoint modules $M, M'\in \mathbb {M}(G)$ are *adjacent* if each vertex of *M* is adjacent to all vertices of $M'$ in *G*; the modules are *non-adjacent* if none of the vertices of *M* is adjacent to a vertex of $M'$ in *G*. By definition of modules, every two disjoint modules $M, M'\in \mathbb {M}(G)$ are either adjacent or non-adjacent [63, Lemma 4.11]. One can therefore define the *quotient graph* $G/\mathbb {M}_{\max }(G)$ which has $\mathbb {M}_{\max }(G)$ as its vertex set and $\{M_i,M_j\}\in E(G/\mathbb {M}_{\max }(G))$ if and only if $M_i$ and $M_j$ are adjacent in *G*. For later reference, we provide the following simple result.

#### Observation 4.2

([56]) The quotient graph $G/\mathbb {M}_{\max }(G)$ with $\mathbb {M}_{\max }(G) = \{M_1, \dots , M_k\}$ is isomorphic to any subgraph induced by a set $W\subseteq V$ such that $|M_i \cap W | = 1$ for all $i \in \{1, \dots ,k\}$.

On a related note, the following lemma provides additional insight into the structure of induced primitive subgraphs in relation to the strong modules of *G*.

#### Lemma 4.3

( [71, L. 3.4], [39, L. 2.5] & [72, Thm. 2.17]) Let *H* be an induced primitive subgraph of *G* and $M\in \mathbb {M}_{\textrm{str}}(G)$ be a strong module that is inclusion-minimal w.r.t. $V(H)\subseteq M$. Then, *M* is prime and *H* is isomorphic to an induced subgraph of $G[M]/\mathbb {M}_{\max }(G[M])$. In particular, $G[M]/\mathbb {M}_{\max }(G[M])$ is primitive for all non-trivial prime modules *M* of *G*. Moreover, every prime module is strong.

In the following, we explore the relationships between modules in graphs and clusters in 0/1-labeled networks that explain them.

#### Lemma 4.4

Let (*N*, *t*) be a 0/1-labeled network with the 2-lca property and consider some non-empty subset $\mathfrak {C}'\subseteq \mathfrak {C}_N$. If $M{:}{=}\cup _{C\in \mathfrak {C}'}C$ overlaps with no cluster in $\mathfrak {C}_N$, then *M* is a module of $\mathscr {G}(N,t)$.

#### Proof

Let (*N*, *t*) be a 0/1-labeled network on *X* with the 2-lca property. Hence $G{:}{=}\mathscr {G}(N,t)$ is well-defined. Assume that $\mathfrak {C}'\subseteq \mathfrak {C}_N$ is a nonempty collection of clusters such that $M{:}{=}\bigcup _{C\in \mathfrak {C}'} C$ overlaps with no cluster in $\mathfrak {C}_N$. If $| M|\in \{1,|X|\}$, then *M* is a trivial module of *G*. Thus, assume henceforth that $1<| M|<|X|$. In particular, there exists a vertex $x\in X\setminus M$ and two distinct vertices $y,y'\in M$. Moreover, since *N* has the 2-lca property, the vertices $u{:}{=}\operatorname {lca}_N(x,y)$ and $u'{:}{=}\operatorname {lca}_N(x,y')$ are well-defined. Since $x,y\in \texttt{C}_N(u)$, and $y\in M$ and $x\notin M$, the fact that *M* does not overlap with $\texttt{C}_N(u)$ implies that $M\subseteq \texttt{C}_N(u)$. Since $y'\in M$, it additionally follows that $\{x,y'\}\subseteq \texttt{C}_N(u)$. Therefore Lemma 2.3 implies that $\operatorname {lca}_N(x,y')\preceq _N u$ i.e. $u'\preceq _N u$. By analogous arguments, one shows that $u\preceq _N u'$. Hence, $u=u'$. Since $x\in X{\setminus } M$ and $y,y'\in M$ have been arbitrarily chosen it follows that $\operatorname {lca}_N(x,y)=\operatorname {lca}_N(x,y')$ and, therefore, $t(\operatorname {lca}_N(x,y))=t(\operatorname {lca}_N(x,y'))$ for all $y,y'\in M$ and all $x\notin M$. This and the fact that (*N*, *t*) explains *G*, implies that *M* is a module of *G*. $\square$

As a simple consequence of Lemma 4.4, we obtain

#### Corollary 4.5

Let (*N*, *t*) be a 0/1-labeled network with the 2-lca property. If there is a vertex *v* such that $\texttt{C}_N(v)$ overlaps with no cluster in $\mathfrak {C}_N$, then $\texttt{C}_N(v)$ is a module of $\mathscr {G}(N,t)$.

**Fig. 5:**
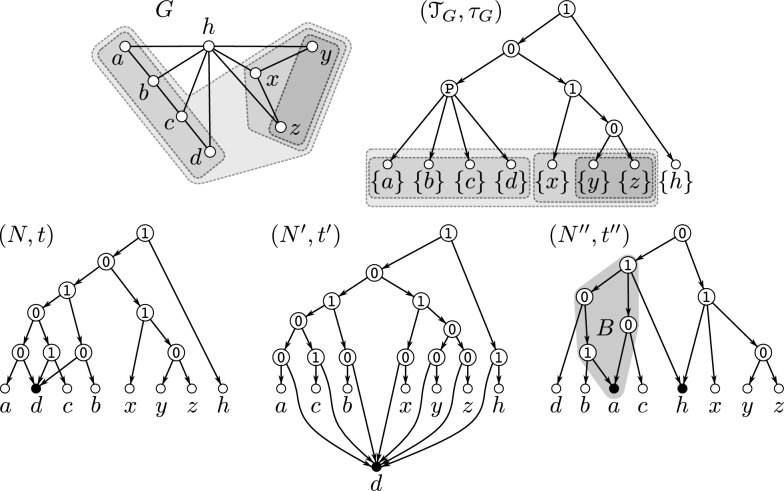
Shown is a graph *G* that is explained by several 0/1-labeled level-1 networks: (*N*, *t*), $(N',t')$, and $(N'',t'')$. Hybrid vertices within these networks are indicated as solid black vertices. The strong modules of *G* are *V*(*G*), the singletons, $\{a,b,c,d,x,y,z\}$, $\{a,b,c,d\}$, $\{x,y,z\}$, and $\{y,z\}$; the latter four are highlighted in gray-shaded areas. Note that *G* is a near-cograph as $G-d$ is a cograph. Here, (*N*, *t*) is a pvr-network obtained from the MDT $(\mathscr {T}_G,\tau _G)$ of *G* by replacing the $\textrm{prime}(\mathtt P)$-labeled vertex by a 0/1-labeled level-1 network according to Definition 4.16. Moreover, according to Definition 3.7 and Eq. (3), $(N',t') = (\tilde{T}_{{\nwarrow }d}, \tilde{t}_{{\nwarrow }d})$ with $(\tilde{T}, \tilde{t})$ being the cotree of $G-d$. In contrast, the network $(N'',t'')$ is not obtained from the MDT of *G* resp. cotree of $G-z$ in the manner as the other two networks. Since the cluster $\{y,z\} \in \mathfrak {C}_{N''}$ overlaps no cluster in $\mathfrak {C}_{N''}$, Corollary 4.5 implies that $\{y,z\}$ is a module of *G*. The same is true for the set $\{x,y,z\}$, which is a (strong) module obtained as the union of clusters $\{x\} \cup \{y,z\}$, which does not overlap with any cluster in $\mathfrak {C}_{N''}$ According to Lemma 4.6, another module of *G* is given by ${{\,\textrm{L}\,}}(B) = \{a,b,c,d\} \subsetneq \texttt{C}_{N''}(\max _B)$ for the non-trivial block *B* of $N''$ (highlighted in gray-shaded area). Note that $\texttt{C}_{N''}(\max _B) = \{a,b,c,d,h\} \in \mathfrak {C}_{N''}$ is not a module of *G* as *h* is adjacent to *x* but none of the vertices *a*, *b*, *c*, *d* is adjacent to *x*

Lemma 4.4 and Corollary 4.5 are illustrated in Fig. 5. In general, $\mathbb {M}_{\textrm{str}}(G)$ is a proper subset of $\mathbb {M}(G)$, that is, *G* can contain overlapping modules. In particular, the module *M* defined in Lemma 4.4 is not necessarily strong. As an example, consider the network $(N'',t'')$ shown in Fig. 3 where any union of clusters $\cup _{i\in I}\{a_i,b_i,c_i,d_i\}$ forms a module in $G = \mathscr {G}(N,t)$. However, for example, $\{a_1,b_1,c_1,d_1,a_2,b_2,c_2,d_2 \}$ and $\{a_2,b_2,c_2,d_2,a_3,b_3,c_3,d_3\}$ overlap and are therefore not strong.

Note that the converse of Lemma 4.4 is, in general, not satisfied. That is, even though every module *M* of $G = \mathscr {G}(N,t)$ can be expressed as the union of clusters (namely, as $M=\cup _{x\in M}\{x\}$) there may exist a cluster $C\in \mathscr {C}_N$ such that *M* and *C* overlap. As an example, consider the graph $G = \mathscr {G}(N'',t'')$ and the modular decomposition tree $(\mathscr {T}_{G}, \tau _{G})$ of *G* as shown in Fig. 5. Here, $M {:}{=} \{a,b,c,d,x,y,z\}$ corresponds to a vertex in $(\mathscr {T}_{G}, \tau _{G})$, namely the 0-labeled child of $\rho _{\mathscr {T}_{G}}$. Thus, *M* is a strong module. However, *M* overlaps with the cluster $\texttt{C}_{N''}(\max _B) = \{a,b,c,d,h\}$ in $N''$.

We can obtain additional modules of $G = \mathscr {G}(N,t)$ by considering the union of leaf-descendants of the vertices $v\ne \max _B$ in a non-trivial block *B*, as follows. Let *B* be a nontrivial block of a network *N*. By Lemma 2.5, *B* has a unique $\preceq _N$-maximal element $\max _B$. Put

$$\begin{aligned} {{\,\textrm{L}\,}}(B)&{:}{=}\{ x\in X\,:\, \text { there is some } \\  &\hspace{0.75cm} v\in V(B)\setminus \{ \textrm{max}_B \} \text { s.t. } x\in \texttt{C}_N(v)\}\\  &=\bigcup _{v\in V(B)\setminus \{\max _B\}}\texttt{C}_N(v). \end{aligned}$$

By Lemma 2.3, ${{\,\textrm{L}\,}}(B)\subseteq \texttt{C}_N(\max _B)$ holds but, as shown in Fig. 5, there are cases when ${{\,\textrm{L}\,}}(B)\ne \texttt{C}_N(\max _B)$.

#### Lemma 4.6

For every nontrivial block *B* of a 0/1-labeled network (*N*, *t*) with the 2-lca property, the set ${{\,\textrm{L}\,}}(B)$ is a module of $\mathscr {G}(N,t)$. If, additionally, *N* is 2-lca-relevant then $|{{\,\textrm{L}\,}}(B)|>1$.

#### Proof

Let (*N*, *t*) be a 0/1-labeled network on *X* with the 2-lca property. Hence $G{:}{=}\mathscr {G}(N,t)$ is well-defined. By definition, $V(G)=X$ holds. Now consider any nontrivial block *B* of *N*. By Lemma 2.5, *B* has a unique $\preceq _N$-maximal element $\max _B$ and, by Lemma 2.3, ${{\,\textrm{L}\,}}(B)\subseteq \texttt{C}_N(\max _B)$ holds.

Assume, for contradiction, that ${{\,\textrm{L}\,}}(B)$ overlaps with some $C\in \mathfrak {C}_N$ and let $v\in V(N)$ be a vertex that satisfies $\texttt{C}_N(v) =C$. In this case, ${{\,\textrm{L}\,}}(B)\cap \texttt{C}_N(v)\ne \emptyset$ implies that there is a vertex $w\in V(B)\setminus \{\max _B\}$ such that $\texttt{C}_N(w) \cap \texttt{C}_N(v)\ne \emptyset$. We distinguish now between the cases that *v* and *w* are $\preceq _N$-incomparable or not. Assume first that *v* and *w* are $\preceq _N$-incomparable. Lemma 2.5 implies that *w* and *v* are located in a common block $B'$. Observe that $w\ne \max _{B'}$ since, otherwise, $v\preceq _N w$ would hold. Hence, $w\notin \{\max _B,\max _{B'}\}$ and Lemma 2.5 implies that $B=B'$. Now there are two cases for *v*; either $v=\max _B$ or not. If $v=\max _B$, then $w\preceq _N v$; a contradiction. If $v\ne \max _B$, then, by definition, $\texttt{C}_N(v)\subseteq {{\,\textrm{L}\,}}(B)$ and ${{\,\textrm{L}\,}}(B)$ does not overlap with $\texttt{C}_N(v)$; a contradiction. Hence, the case that *v* and *w* are $\preceq _N$-incomparable cannot occur.

Assume now that *v* and *w* are $\preceq _N$-comparable. If $v\preceq _N w$, Lemma 2.3 implies that $\texttt{C}_N(v)\subseteq \texttt{C}_N(w)\subseteq {{\,\textrm{L}\,}}(B)$; contradicting the fact that $\texttt{C}_N(v)$ and ${{\,\textrm{L}\,}}(B)$ overlap. Hence, it must hold that $w\prec _N v$. If $\max _B \preceq _N v$, Lemma 2.3 implies that we have ${{\,\textrm{L}\,}}(B) \subseteq \texttt{C}_N(\max _B) \subseteq \texttt{C}_N(v)$; again a contradiction. If $v \prec _N \max _B$, there is a directed path from $\max _B$ to *w* that contains *v* and each such path is, by Lemma 2.5, completely contained in *B*. Thus, $v\in V(B)$. But then $v\ne \max _B$ implies that $\texttt{C}_N(v)\subseteq {{\,\textrm{L}\,}}(B)$; again a contradiction. Therefore, *v* and $\max _B$ must be $\preceq _N$-incomparable. Since ${{\,\textrm{L}\,}}(B) \subseteq \texttt{C}_N(\max _B)$ it follows that $\texttt{C}_N(v)\cap \texttt{C}_N(\max _B)\ne \emptyset$ and, by Lemma 2.5, *v* and $\max _B$ are contained in some common non-trivial block $B'$. Hence, there is a directed path $P_{\max _{B'}v}$ from $\max _{B'}$ to *v* and a directed path $P_{\max _{B'}\max _B}$ from $\max _{B'}$ to $\max _B$. Note that $P_{\max _{B'}v}$ does not contain $\max _B$ and that $P_{\max _{B'}\max _B}$ does not contain *v*, since *v* and $\max _B$ are $\preceq _N$-incomparable. Let now $w''$ be the $\preceq _N$-minimal vertex that is contained in $P_{\max _{B'}v}$ and $P_{\max _{B'}\max _B}$ and denote with $P_{w''v}$ and $P_{w''\max _B}$ the subpath of $P_{\max _{B'}v}$ from $w''$ to *v* and the subpath of $P_{\max _{B'}\max _B}$ from $w''$ to $\max _B$, respectively. By construction, $P_{w''v}$ and $P_{w''\max _B}$ have only the vertex $w''$ in common. Since $w\prec _N v$ and $w\prec _N \max _B$, there is a directed *vw*-path $P_{vw}$ and directed $\max _B w$-path $P_{\max _Bw}$. Let $w'$ be the $\preceq _N$-maximal vertex that is contained in $P_{vw}$ and $P_{\max _Bw}$ and denote with $P_{vw'}$ and $P_{\max _B w'}$ the subpath of $P_{vw}$ from *v* to $w'$ and the subpath of $P_{\max _Bw}$ from $\max _B$ to $w'$, respectively. By construction, $P_{vw'}$ and $P_{\max _B w'}$ have only the vertex $w'$ in common. Since $w'\prec _N v$ and *v* and $\max _B$ are $\preceq _N$-incomparable, we have $w'\ne \max _B$. In particular, $w'\in V(B)\setminus \{\max _B\}$, since $w'$ is a vertex of the path $P_{\max _Bw}$ which, by Lemma 2.5, is completely contained in *B*. Since *N* is a DAG, we can now construct a directed path *P* by combining $P_{w''v}$ with $P_{vw'}$ and a directed path $P'$ by combining $P_{w''\max _B}$ with $P_{\max _Bw'}$ to obtain two directed paths that have only the vertices $w'$ and $w''$ in common. In particular, the subgraph of *N* induced by the vertices of *P* and $P'$ is biconnected and is thus contained in some non-trivial block $B''$. By construction, $B''$ contains $v, w'$ and $w''$. This and $w'\prec _N w''$ implies that $w'\in V(B'')\setminus \{\max _{B''}\}$. As argued above, $w'\in V(B)\setminus \{\max _B\}$. Thus, we can apply Lemma 2.5 to conclude that $B=B''$. Since $v\in V(B'')$, we have $v\in V(B)$. Therefore, $v\preceq _N \max _B$ must hold; a contradiction. In summary, ${{\,\textrm{L}\,}}(B)$ cannot overlap with any cluster $C\in \mathfrak {C}_N$. By Lemma 4.4, ${{\,\textrm{L}\,}}(B)$ is a module of $\mathscr {G}(N,t)$.

For the second statement, furthermore assume that *N* has the 2-lca property and is 2-lca-relevant. Since *B* is a non-trivial block, there exist vertices $w,v\in V(B)$ such that $w\prec _N v\prec _N \max _B$. Moreover, Lemma 2.3 implies that $\texttt{C}_N(w)\subseteq \texttt{C}_N(v)$. Since *N* is 2-lca-relevant, *v* is a least common ancestor for some subset $A\subseteq X$ of size $|A|\le 2$. Thus, $\texttt{C}_N(w) = \texttt{C}_N(v)$ is not possible as, otherwise, $w\prec _N v$ implies that *v* is not a least common ancestor for any subset $A\subseteq X$. Hence, $\texttt{C}_N(w) \subsetneq \texttt{C}_N(v)$ holds. Therefore, $|\texttt{C}_N(v)|>1$ which, together with $\texttt{C}_N(v)\subseteq {{\,\textrm{L}\,}}(B)$, implies that $|{{\,\textrm{L}\,}}(B)|>1$ $\square$

### Characterizing primitive Lev-1-Ex graphs

We explore now the structure of primitive Lev-1-Ex graphs. First, we show that any primitive Lev-1-Ex graph is explained by a 0/1-labeled level-1 network with fairly constrained structural properties. We then prove that primitive Lev-1-Ex graphs are precisely the primitive near-cographs, as defined below.

#### Lemma 4.7

If *G* is a primitive Lev-1-Ex graph, then there exists a 0/1-labeled phylogenetic level-1 network (*N*, *t*) that explains *G* and that contains exactly one hybrid and this hybrid is a leaf. In particular, *N* contains precisely one non-trivial block.

#### Proof

Let *G* be a primitive graph and assume that (*N*, *t*) is a 0/1-labeled level-1 network that explains *G*. By definition, *N* has the 2-lca property. Moreover, by Theorem 3.6, we can assume w.l.o.g. that *N* is a 2-lca-relevant and regular level-1 network. By Lemma 2.10, *N* is phylogenetic. Since *G* is primitive, it cannot be a cograph and Theorem 3.3 implies that *G* cannot be explained by a tree. Thus, *N* must contain at least one non-trivial block *B*. By Lemma 4.6, the set ${{\,\textrm{L}\,}}(B)$ is a module of *G* and $|{{\,\textrm{L}\,}}(B)|>1$. Since *G* is primitive, we must thus have that ${{\,\textrm{L}\,}}(B)=X$. Moreover, Lemma 2.5 implies that $v\preceq _N \max _B$ for all $v\in V(B)$. This together with Lemma 2.3 implies that ${{\,\textrm{L}\,}}(B)\subseteq \texttt{C}_N(\max _B)$. Hence, $\texttt{C}_N(\max _B)=X$. Since $\texttt{C}_N(\rho _N)=X$ and every vertex of a regular network is associated with a unique cluster (cf. Lemma 2.10), it follows that $\max _B=\rho _N$.

Assume, for contradiction, that there is a non-trivial block $B'$ of *N* such that $B'\ne B$. By applying the same arguments as for *B*, we can conclude that ${{\,\textrm{L}\,}}(B')=X$ and $\max _{B'}=\rho _N$. Let $x\in X$. Thus, there are vertices $u\in V(B)\setminus \{\max _{B}\}$ and $v\in V(B')\setminus \{\max _{B'}\}$ such that $x\in \texttt{C}_N(u)\cap \texttt{C}_N(v)$. Observe first that $u\preceq _N v$ is not possible since, otherwise, there is a directed $\rho _Nu$-path in *N* containing *v* and each such path is by Lemma 2.5 completely contained in *V*(*B*), which contradicts $v\notin V(B)$. Similarly, $v\preceq _N u$ cannot occur. Hence, *u* and *v* are $\preceq _N$-incomparable. Lemma 2.5 implies that *u* and *v* are contained in a common non-trivial block; a contradiction to $B\ne B'$. Hence, there is no non-trivial block $B'$ of *N* such that $B'\ne B$. In particular, all hybrid vertices of *N* belong to some non-trivial block and since there is only one, namely *B*, all hybrid vertices of *N* are vertices of *B*. Since *N* is level-1 there is thus a unique hybrid vertex *h* in *N*.

Consider now the unique hybrid *h* and its cluster $\texttt{C}_N(h)$. Assume first that there exists a vertex *v* in *N* such that $\texttt{C}_N(v)$ and $\texttt{C}_N(h)$ overlap. Then, by [36, L. 18 & L. 19], there is a hybrid vertex $h'$ in *B* such that $h'\prec _N h$; contradicting the uniqueness of *h*. Hence, $\texttt{C}_N(h)$ does not overlap with any cluster in $\mathfrak {C}_N$. This and Corollary 4.5 implies that $\texttt{C}_N(h)$ is a module of *G*. Since *G* is primitive, $\texttt{C}_N(h)$ must be a trivial module. Since $h\ne \rho _N$, Lemma 2.10 ensures that $\texttt{C}_N(h)\ne \texttt{C}_N(\rho _N)=X$. Thus, $\texttt{C}_N(h)=\{x\}$ for some $x\in X$. Since $\texttt{C}_N(h) = \{x\}= \texttt{C}_N(x)$ and due to Lemma 2.10, we can conclude that $h=x$ i.e. that *h* is a leaf. $\square$

#### Definition 4.8

A graph $G=(V,E)$ is a *near-cograph* if $|V|=1$ or if there is a vertex $v\in V$ such that $G-v$ is a cograph.

By Theorem 3.3, the property of being a cograph is hereditary. Hence, every cograph is, in particular, a near-cograph. In addition, a cograph does not contain induced $P_4$s. Thus, we obtain

#### Observation 4.9

A graph *G* is near-cograph if and only if *G* contains a vertex that is located on all induced $P_4$s in *G* if there are any.

Next, we provide a simple characterization of near-cographs that, in particular, shows that near-cographs form a hereditary graph class that is closed under complementation.

#### Lemma 4.10

The following statements are equivalent.

1. *G* is a near-cograph.
2. $\overline{G}$ is a near-cograph.
3. Every induced subgraph of *G* is a near-cograph.

#### Proof

The equivalences are trivial for single-vertex graphs. Hence, suppose that $G = (V,E)$ is a graph with $|V|>1$. If *G* is a near-cograph, then $G-v$ is a cograph for some $v\in V(G)=V({\overline{G}})$. By Theorem 3.3, the complement of a cograph is a cograph and it follows that $\overline{G-v} = \overline{G}-v$ is a cograph. Hence, ${\overline{G}}$ is a near-cograph. Similar arguments show that *G* is a near-cograph whenever $\overline{G}$ is, i.e., (1) and (2) are equivalent.

Suppose now that *G* is a near-cograph. Assume, for contradiction, that *G* contains an induced subgraph *H* that is not a near-cograph. By Observation 4.9, *H* contains no vertex that is located on all induced $P_4$s. Since *H* is an induced subgraph of *G*, this property remains in *G* and Observation 4.9 implies that *G* is not a near-cograph; a contradiction. Hence, (1) implies (3). Moreover, (3) implies (1) since $H=G$ is an induced subgraph of *G* and thus, a near-cograph. $\square$

As an immediate consequence of Theorem 3.10 and the definition of near-cographs, we also obtain the following

#### Corollary 4.11

Every near-cograph is Lev-1-Ex.

The converse of Corollary 4.11 does not hold; consider, for example, the Lev-1-Ex graph *G* in Fig. 3, consisting of $k > 1$ vertex-disjoint $P_4$s. By Observation 4.9, this implies that *G* is not a near-cograph. Despite this, we are now in a position to provide a characterization of primitive Lev-1-Ex graphs.

#### Theorem 4.12

Let *G* be a primitive graph. The following statements are equivalent:

- *G* is a near-cograph.
- *G* is Lev-1-Ex.

#### Proof

Let $G=(X,E)$ be a primitive graph. First assume that *G* is a near-cograph. Hence, there is a vertex $v\in X$ such that $G-v$ is a cograph. By Theorem 3.10, *G* is ${\textsc {Lev}}\hbox {-}{|\{v\}|}\hbox {-}{\textsc {Ex}}$, that is, Lev-1-Ex.

Conversely, assume that *G* is Lev-1-Ex. Since *G* additionally is primitive, Lemma 4.7 implies that there exists a 0/1-labeled phylogenetic level-1 network (*N*, *t*) on *X* that explains *G* such that *N* contains exactly one hybrid $h\in X$ and exactly one non-trivial block *B*. By definition of primitivity, $|X|\ge 4$ and thus, $X{\setminus } \{h\}\ne \emptyset$.

We show now that $\mathfrak {C}'{:}{=} \mathfrak {C}_N\cap (X\setminus \{h\})$ is a hierarchy. Observe first that $X{\setminus } \{h\}\in \mathfrak {C}'$ and $\{x\} \in \mathfrak {C}'$ for all $x\in X{\setminus } \{h\}$, which ensures that $\mathfrak {C}'$ is a clustering system. Assume, for contradiction, that there are overlapping clusters $A, B \in \mathfrak {C}'$ and put $C {:}{=} A\cap B$. The clusters *A* and *B* stem from two clusters $\texttt{C}_N(u), \texttt{C}_N(v) \in \mathfrak {C}$ via $A = \texttt{C}_N(u){\setminus } \{h\}$ and $B = \texttt{C}_N(v){\setminus } \{h\}$. Note that $A = \texttt{C}_N(u)$ or $B = \texttt{C}_N(v)$ may be possible. Since $A\cap B = C\ne \emptyset$ and $h\notin C$ it follows that $\texttt{C}_N(u)\cap \texttt{C}_N(v)\ne \emptyset$ and $\texttt{C}_N(u)\cap \texttt{C}_N(v)\ne \{h\}$. Since *h* is the only hybrid vertex in *N*, Lemma 2.9 implies that $\texttt{C}_N(v)\subseteq \texttt{C}_N(u)$ or $\texttt{C}_N(u)\subseteq \texttt{C}_N(v)$ holds. We may assume w.l.o.g. that $\texttt{C}_N(u)\subseteq \texttt{C}_N(v)$. If $h\in \texttt{C}_N(u)$, then $h\in \texttt{C}_N(v)$ and we obtain $A\subseteq B$; a contradiction. If $h\notin \texttt{C}_N(u)$, then $A=\texttt{C}_N(u) \subseteq B$; again a contradiction. Thus, there are no overlapping $A, B \in \mathfrak {C}'$. Consequently, $\mathfrak {C}'$ is a hierarchy.

Since $\mathfrak {C}'$ is a hierarchy, Theorem 2.11 ensures that $\mathscr {H}(\mathfrak {C}')$ is a phylogenetic tree. By Proposition 3.5, $T\doteq \mathscr {H}(\mathfrak {C}')$ can be equipped with a 0/1-labeling $t'$ such that $(T,t')$ explains $G[X\setminus \{h\}] = G-h$. By Theorem 3.3, $G-h$ is a cograph and, therefore, *G* is a near-cograph. $\square$

An alternative proof strategy for the “(b) $\Rightarrow$ (a)” direction could be to consider the network (*N*, *t*) on *X* that explains $G=(X,E)$, where *N* contains exactly one hybrid $h \in X$ and exactly one non-trivial block *B*. By removing *h* and its incident edges from *N*, we obtain a 0/1-labeled tree $(T,t')$ on $X\setminus \{h\}$. It then suffices to show that $(T,t')$ explains $G-h$ to verify that $G-h$ is a cograph. Instead of showing that, for all $x, y \in X{\setminus } \{h\}$, it holds that $\operatorname {lca}_T(x,y) = 1$ if and only if $\{x,y\} \in E(G-h)$ we used the current proof strategy which relies solely on the clustering system of *N*.

**Fig. 6:**
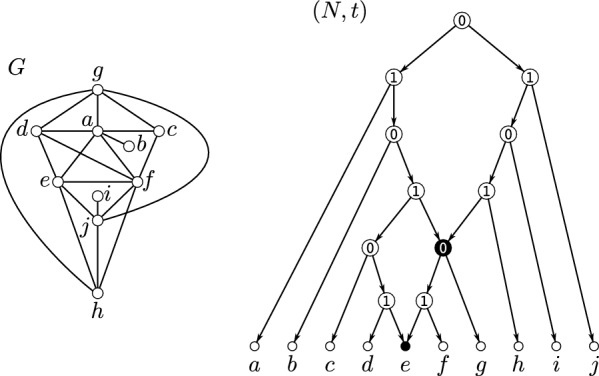
A primitive graph $G=\mathscr {G}(N,t)$ that is explained by the 0/1-labeled level-2 network (*N*, *t*) whose hybrid vertices are indicated as solid black vertices. Here, there is no subset $Y\subseteq V(G)$ of size $|Y|\le 2$ such that $G-Y$ is a cograph. To see this, assume, for contradiction, that there is a set *Y* of size $|Y|\le 2$ resulting in the cograph $G-Y$. First observe that there are two intertwined induced $P_4$s $G[\{b,a,f,j\}]$ and $G[\{a,f,j,i\}]$ that are both vertex-disjoint with the induced $P_4$ $G[\{c,g,d,e\}]$. Hence, *Y* must contain one vertex of *a*, *f*, *j* and one vertex of *c*, *g*, *d*, *e* to destroy these $P_4$. If $j\in Y$, then $Y = \{j,v\}$ with $v\in \{c,g,d,e\}$ would leave the induced $P_4$ $G[\{b,a,f,h\}]$ in $G-Y$. Hence, $j\notin Y$. If $f\in Y$ and $Y = \{f,e\}$, then the induced $P_4$ $G[\{b,a,g,j\}]$ is in $G-Y$. Thus, $Y\ne \{f,e\}$ and, if $f\in Y$ then, $Y = \{f,v\}$ with $v\in \{c,g,d\}$ must hold in which case $G-Y$ contains the induced $P_4$ $G[\{a,e,j,i\}]$. Thus, $f\in Y$ is not possible. Thus, $a\in Y$ must hold. If $Y=\{a,g\}$ or $Y=\{a,c\}$, the induced $P_4$ $G[\{d,e,j,i\}]$ remains in $G-Y$. If $Y=\{a,d\}$ or $Y=\{a,e\}$, the induced $P_4$ $G[\{c,f,j,i\}]$ remains in $G-Y$. Thus, none of the combinations $Y=\{v,w\}$ with $v\in \{a,f,j\}$ $w\in \{c,g,d,e\}$ yield a cograph. Thus, there is no set *Y* of size $|Y|\le 2$ resulting in the cograph $G-Y$

Note that Theorem 4.12 cannot be generalized to primitive Lev-*k*-Ex graphs. As an example, consider the primitive Lev-2-Ex graph *G* shown in Fig. 6 where it is necessary to remove at least three vertices from *G* in order to obtain a cograph.

### Characterizing general Lev-1-Ex graphs

In this section, we characterize Lev-1-Ex graphs. As the following result shows, the property of being a clustering system that is closed or satisfies (L) is hereditary. This fact, in turn, enables us to prove that the property of a graph being Lev-1-Ex is also hereditary.

#### Lemma 4.13

( [38, L. 2.1]) Let $\mathfrak {C}$ be a clustering system on *X* with $|X|>1$ and let $x\in X$. Then, $\mathfrak {C}-x$ is a clustering system on $X\setminus \{x\}$. If $\mathfrak {C}$ satisfies property $\Pi \in \{closed, \text {(L)}\}$, then $\mathfrak {C}-x$ satisfies $\Pi$.

#### Proposition 4.14

A graph *G* is Lev-1-Ex if and only if *G*[*W*] is Lev-1-Ex for all non-empty subsets $W\subseteq V(G)$.

#### Proof

The *if* direction follows from the fact that *G*[*W*] is Lev-1-Ex for $W= V(G)$ and that, in this case, $G[W]= G$. Suppose now that $G=(X,E)$ is Lev-1-Ex and thus, explained by a 0/1-labeled level-1 network (*N*, *t*) on *X*. Let $W\subseteq X$ be non-empty. Put $\mathfrak {C}'{:}{=} \mathfrak {C}_N\cap W = \{C\cap W :C\in \mathfrak {C}_N \text { and } C \cap W\ne \emptyset \}$. Since *N* is a level-1 network, Theorem 2.12 implies that $\mathfrak {C}_N$ is closed and satisfies (L). Note that for ${\overline{W}}=\{x_1,\ldots ,x_\ell \}= X\setminus W$ it holds that $C\cap W=C{\setminus } {\overline{W}}=(\ldots ((C{\setminus }\{x_1\}){\setminus }\{x_2\})\ldots ){\setminus }\{x_\ell \}$ for each $C\in \mathfrak {C}_N$. Thus $\mathfrak {C}'$ can be obtained as $\mathfrak {C'}=(\ldots ((\mathfrak {C}_N-x_1)-x_2)\ldots )-x_\ell$. Repeated application of Lemma 4.13 to all $x\in X\setminus W$ shows that $\mathfrak {C}'$ must be closed and satisfy (L). Theorem 2.12 implies that $N'\doteq \mathscr {H}(\mathfrak {C}')$ is a level-1 network on *W*. By Lemma 2.8, $N'$ is an lca-network. This together with Proposition 3.5 implies that $N'$ can be equipped with a 0/1-labeling $t'$ such that $(N',t')$ explains *G*[*W*]. In summary, *G*[*W*] is Lev-1-Ex. $\square$

Our goal is to modify the MDT of a Lev-1-Ex graph *G* to construct a 0/1-labeled level-1 network that explains *G*. To achieve this, we first introduce the concept of “prime vertex replacement”, as originally defined in [52] and subsequently utilized in [37–39]. In this work, we adhere to the terminology and definitions established in [38].

#### Definition 4.15

(**prime-explaining family**) Let $G = (X,E)$ be a graph with modular decomposition tree $(\mathscr {T},\tau )$ and $\mathbb {P}\subseteq \mathbb {M}_{\textrm{str}}(G)$ be the set of all of its non-trivial prime modules. Two networks *N* and $N'$ are *internal vertex-disjoint* if $(V^0(N)\setminus \{\rho _N\})\cap (V^0(N')\setminus \{\rho _{N'}\})=\emptyset$. A *prime-explaining family* (*of G)* is a set $\mathcal {P}(G)=\{(N_M,t_M) :M\in \mathbb {P}\}$ of pairwise internal vertex-disjoint 0/1-labeled networks such that, for each $M\in \mathbb {P}$, the network $(N_M,t_M)$ explains the quotient graph $G[M]/\mathbb {M}_{\max }(G[M])$.

As we shall see, the modular decomposition tree $(\mathscr {T},\tau )$ of any graph *G* can be modified by locally replacing prime vertices $M \in \mathbb {P}$ with $(N_M, t_M) \in \mathcal {P}(G)$ to construct a 0/1-labeled network that explains *G*. This concept is formalized in the following definition.

#### Definition 4.16

(**prime-vertex replacement (pvr) networks**) Let $G=(X, E)$ be a graph and $\mathbb {P}$ be the set of all non-trivial prime vertices in its modular decomposition tree $(\mathscr {T},\tau )$. Let $\mathcal {P}(G)$ be a prime-explaining family of *G*. A *prime-vertex replacement (pvr) network* ${{\,\textrm{pvr}\,}}(G,\mathcal {P}(G))$ of *G* is the directed, 0/1-labeled graph (*N*, *t*) obtained by the following procedure:

1. For each $M\in \mathbb {P}$, remove all edges $(M,M')$ with $M'\in {{\,\textrm{child}\,}}_{\mathscr {T}}(M)$ from $\mathscr {T}$ to obtain the directed graph $T'$.
2. Construct a directed graph *N* by adding, for each $M\in \mathbb {P}$, $N_M$ to $T'$ by identifying the root of $N_M$ with *M* in $T'$ and each leaf $M'$ of $N_M$ with the corresponding child $M'\in {{\,\textrm{child}\,}}_{\mathscr {T}}(M)$.
3. Define the labeling $t:V^0(N)\rightarrow \{0,1\}$ by putting, for all $w\in V^0(N)$,
  $$\begin{aligned} t(w) {:}{=} {\left\{ \begin{array}{ll} t_{M}(w) & \text{ if } w \in V^0(N_{M}) \text { for some } M\in \mathbb {P}\\ \tau (w) & \text{ otherwise, } \text{ i.e., } \text{ if } w\in V^0(\mathscr {T})\setminus \mathbb {P}\end{array}\right. } \end{aligned}$$
4. Replace every singleton module $\{x\}\in V(N)$ in *N* by *x*.

The resulting 0/1-labeled directed graph (*N*, *t*) is called a *pvr-network* (*of G)* and denoted by ${{\,\textrm{pvr}\,}}(G,\mathcal {P}(G))$.

The following proposition establishes that the pvr-network provides a 0/1-labeled network explaining any graph *G*, given a prime-explaining family $\mathcal {P}(G)$. In Lemma 4.18, which generalizes [38, L. 6.19], we further show that pvr-networks preserve certain properties of the networks included in $\mathcal {P}(G)$. In particular, the level of the pvr-network corresponds to the maximum level of the networks in $\mathcal {P}(G)$.

#### Proposition 4.17

([38, L. 5.3 & Prop. 5.5]) For every graph $G=(X,E)$, the pvr-network ${{\,\textrm{pvr}\,}}(G,\mathcal {P}(G))$ is a 0/1-labeled network on *X* that explains *G*, for all prime-explaining families $\mathcal {P}(G)$.

#### Lemma 4.18

Let $\mathcal {P}(G)$ be a prime-explaining family of a graph *G*. If all 0/1-labeled networks in $\mathcal {P}(G)$ are phylogenetic, then the pvr-network ${{\,\textrm{pvr}\,}}(G,\mathcal {P}(G))$ is phylogenetic. If all 0/1-labeled networks in $\mathcal {P}(G)$ are level-*k*, then ${{\,\textrm{pvr}\,}}(G,\mathcal {P}(G))$ is level-*k*.

#### Proof

Let $\mathcal {P}(G)=\{(N_M,t_M):M\in \mathbb {P}\}$ be a prime-explaining family of the graph $G = (X,E)$, where $\mathbb {P}$ is the set of prime-labeled vertices in the MDT $(\mathscr {T},\tau )$ of *G*. Furthermore, put $(N,t){:}{=}{{\,\textrm{pvr}\,}}(G,\mathcal {P}(G))$. By Proposition 4.17, *N* is a network on *X*.

Assume first that, for each $(N_M,t_M)\in \mathcal {P}(G)$, the network $N_M$ is phylogenetic. Since $\mathscr {T}$ is phylogenetic and the in- and out-degrees of non-prime vertices in $\mathscr {T}$ remain unaffected when constructing *N*, it follows that all vertices *v* in $\mathscr {T}$ that do not correspond to a prime module of *G* do not satisfy ${{\,\textrm{outdeg}\,}}_N(v)=1$ and ${{\,\textrm{indeg}\,}}_N(v)\le 1$. Let us now consider a vertex *v* in $\mathscr {T}$ that corresponds to a prime module $M'$ of *G*. In this case, *v* is now the root of $N_{M'}$ and thus, ${{\,\textrm{outdeg}\,}}_N(v)={{\,\textrm{outdeg}\,}}_{N_{M'}}(\rho _{N_{M'}})>1$ must hold. Moreover, since $N_{M'}$ is phylogenetic, none of its inner vertices *v* satisfy ${{\,\textrm{outdeg}\,}}_N(v)=1$ and ${{\,\textrm{indeg}\,}}_N(v)\le 1$. If, on the other hand, *v* is a leaf in $N_{M'}$, then it corresponds to some vertex in $\mathscr {T}$ which, by the latter arguments, does not satisfy ${{\,\textrm{outdeg}\,}}_N(v)=1$ and ${{\,\textrm{indeg}\,}}_N(v)\le 1$. In summary, *N* is phylogenetic.

Assume now that, for each $(N_M,t_M)\in \mathcal {P}(G)$, the network $N_M$ is level-*k*. To verify that *N* is level-*k* network, we must verify that every non-trivial block *B* of *N* contains at most *k* hybrids distinct from $\max _B$. Observe that, for any two $M',M\in \mathbb {P}$, the networks $N_M$ and $N_{M'}$ can, by construction, share at most one vertex *v*, namely the root of one of these networks and a leaf of the other network. It follows that, in the latter case, *v* is a vertex such that $N-v$ is disconnected. This immediately implies that *B* is a non-trivial block in *N* if and only if *B* is a non-trivial block in $N_M$ for precisely one $M\in \mathbb {P}$. This and the fact that all non-trivial blocks *B* in each of the $N_M$ contain at most *k* hybrids distinct from $\max _B$, implies that every non-trivial block *B* in *N* contains at most *k* hybrids distinct from $\max _B$. Taking the latter arguments together, *N* is a level-*k* network. $\square$

We are, finally, in a position to provide several characterizations of Lev-1-Ex graphs.

#### Theorem 4.19

A graph *G* is Lev-1-Ex if and only if for every non-trivial prime module *M* of *G* the quotient graph $G[M]/\mathbb {M}_{\max }(G[M])$ is a near-cograph.

#### Proof

Let *G* be Lev-1-Ex. If *G* does not contain any non-trivial prime module, then the statement is vacuously true. Thus, suppose that *G* contains a prime module *M* with $|M|>1$. Consider the quotient graph $G'{:}{=} G[M]/\mathbb {M}_{\max }(G[M])$. with $\mathbb {M}_{\max }(G[M]) = \{M_1, \dots , M_k\}$. By Observation 4.2, $G'\simeq G[W]$ with $W\subseteq M$ such that for all $M'\in \mathbb {M}_{\max }(G[M])$ we have $|M' \cap W | = 1$. By Proposition 4.14, $G'$ is Lev-1-Ex since *G* is Lev-1-Ex. Since *M* is a prime module with $|M|>1$, Lemma 4.3 ensures that $G'$ is a primitive graph. The latter two arguments together with Theorem 4.12 imply that $G'$ is a near-cograph.

Now suppose that for every non-trivial prime module *M* of *G* the quotient graph $G_M{:}{=} G[M]/\mathbb {M}_{\max }(G[M])$ is a near-cograph. By Corollary 4.11, $G_M$ is Lev-1-Ex for every non-trivial prime module *M* of *G*. Hence, there exists a prime-explaining family $\mathcal {P}$ of *G* containing only labeled level-1 networks. By Lemma 4.18, the pvr-network $(N,t){:}{=}{{\,\textrm{pvr}\,}}(G,\mathcal {P})$ is a level-1 network and, by Proposition 4.17, (*N*, *t*) explains *G*. Thus, *G* is Lev-1-Ex. $\square$

#### Theorem 4.20

A graph *G* is Lev-1-Ex if and only if all primitive induced subgraphs of *G* are near-cographs.

#### Proof

By contraposition, assume that *G* contains a primitive induced subgraph $H=G[W]$ that is not a near-cograph and let *M* be an inclusion-minimal strong module that contains *W*. By Lemma 4.3, *M* is prime and *H* is isomorphic to an induced subgraph of $G[M]/\mathbb {M}_{\max }(G[M])$. By Lemma 4.10, the property of being a near-cograph is hereditary. The latter arguments imply that $G[M]/\mathbb {M}_{\max }(G[M])$ cannot be a near-cograph. By Theorem 4.19, *G* is not Lev-1-Ex. This establishes the *only-if* direction.

Assume now that all primitive induced subgraphs of *G* (if there are any) are near-cographs. Note that if *G* does not contain prime modules at all, then Theorem 4.19 implies that *G* is Lev-1-Ex. Hence, assume that *G* contains a prime module *M*. By Observation 4.2, $G[M]/\mathbb {M}_{\max }(G[M])\simeq H$ where *H* is an induced subgraph of *G*. In particular, *H* is primitive by Lemma 4.3. By assumption, *H* is a near-cograph. Since the latter argument holds for all prime modules of *G*, Theorem 4.19 implies that *G* is Lev-1-Ex. This establishes the *if* direction. $\square$

Note that primitivity of the induced subgraphs of *G* cannot be omitted in Theorem 4.20. By way of example, the subgraph *G*[*W*] of the graph *G* in Fig. 3 that is induced by $W = \{a_1,b_1,c_1,d_1,a_2,b_2,c_2,d_2\}$ consists of two vertex-disjoint $P_4$s and is, therefore, not a near-cograph even though *G* is Lev-1-Ex.

The latter results can now be used to establish

#### Theorem 4.21

A graph is Lev-1-Ex if and only if it can be explained by a *phylogenetic* 0/1-labeled level-1 network.

#### Proof

The *if* direction is trivial. Suppose that *G* is Lev-1-Ex. If *G* does not contain any non-trivial prime module, then *G* is a cograph and the cotree that explains *G* is a phylogenetic 0/1-labeled level-1 network. Suppose that *G* contains prime modules. Let $\mathfrak {M}$ denote the set of all non-trivial prime modules *M* of *G*. By Theorem 4.19 and Lemma 4.3, the quotient graph $G_M{:}{=} G[M]/\mathbb {M}_{\max }(G[M])$ is a primitive near-cograph for all $M\in \mathfrak {M}$. By Theorem 4.12, $G_M$ is Lev-1-Ex for all $M\in \mathfrak {M}$. By Lemma 4.7, there exists a 0/1-labeled phylogenetic level-1 network $(N_M,t_M)$ that explains $G_M$ for all $M\in \mathfrak {M}$. Hence, there exists a prime-explaining family $\mathcal {P}$ of *G* containing only phylogenetic 0/1-labeled level-1 networks. By Lemma 4.18, the pvr-network ${{\,\textrm{pvr}\,}}(G,\mathcal {P}(G))$ is a phylogenetic 0/1-labeled level-1 network. By Proposition 4.17, ${{\,\textrm{pvr}\,}}(G,\mathcal {P}(G))$ explains *G*. This establishes the *only if* direction. $\square$

### Linear-time algorithm

We now present an algorithm that determines, in linear time, whether a given graph is Lev-1-Ex and, if so, constructs a 0/1-labeled level-1 network explaining the input graph.

**Algorithm 1:**
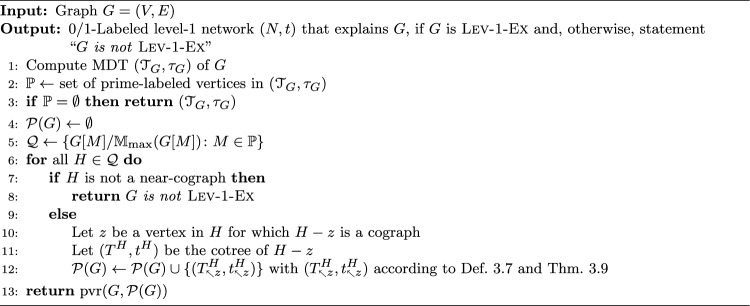
Check if *G* is Lev-1-Ex and construct Level-1 network that explains *G*

#### Theorem 4.22

It can be verified in $O(|X|+|E|)$ time if a given graph $G=(X,E)$ can be explained by a labeled level-1 network and, in the affirmative case, a 0/1-labeled level-1 network (*N*, *t*) that explains *G* can be constructed within the same time complexity.

#### Proof

To prove the statement, we employ Algorithm 1 and start with proving its correctness. Let $G=(X,E)$ denote the input graph. The algorithm first computes the modular decomposition tree $(\mathscr {T}_G,\tau _G)$ of *G* and, in particular, finds the set $\mathbb {P}$ of $\textrm{prime}$-labeled vertices of $\mathscr {T}_G$. If $\mathbb {P}=\emptyset$, then $\tau _G$ is a 0/1-labeling of the tree $\mathscr {T}_G$ and, therefore, *G* is a cograph and $(\mathscr {T}_G,\tau _G)$ is a level-1 network that explains *G* (c.f. Theorem 3.3). Consequently, *G* is Lev-1-Ex and $(\mathscr {T}_G,\tau _G)$ is correctly returned on Line 3. Henceforth, assume that $\mathbb {P}\ne \emptyset$. Next, an empty set $\mathcal {P}(G)$ is initialized and the quotient graph $G[M]/\mathbb {M}_{\max }(G[M])$ is constructed for each $M\in \mathbb {P}$, where $\mathcal {Q}$ comprise all such quotients. Due to Theorem 4.19, *G* is Lev-1-Ex if and only if *H* is a near-cograph for every $H\in \mathcal {Q}$. Therefore, the *for*-loop starting on Line 6 and the *if*-clause on Line 7 will correctly determine whenever *G* is not Lev-1-Ex and thereafter terminate. If, instead, *G* is Lev-1-Ex then Lines 10–12 will run for each $H\in \mathcal {Q}$. More specifically, the cotree $(T^H,t^H)$ of the cograph $H-z$ for some $z\in V(H)$ can be found and it, by definition, explains $H-z$. By Lemma 3.8 and Theorem 3.9, there is a 0/1-labeling $t^H_{{\nwarrow }z}$ such that $(T^H_{{\nwarrow }z},t^H_{{\nwarrow }z})$ is a level-1 network that explains *H*. Consequently, after the last iteration of the loop, the set $\mathcal {P}(G)$ will be a prime-explaining family of *G* containing only level-1 networks. By Proposition 4.17 and Lemma 4.18, ${{\,\textrm{pvr}\,}}(G,\mathcal {P}(G))$ is therefore a level-1 network that explains *G*. This network is, correctly, returned in Line 13.

We now consider the runtime. Computation of the MDT $(\mathscr {T}_G,\tau _G)$ of *G* in Line 1 can be achieved in $O(|X|+|E|)$ time [56]. The number of vertices in $\mathscr {T}_G$ is in *O*(|*X*|) [66] and thus, the set $\mathbb {P}$ of prime vertices in $(\mathscr {T}_G,\tau _G)$ can be determined in *O*(|*X*|) time (Line 2). The tasks in Line 3 and 4 take constant time. As shown in [73, L. 2], *all* quotients $G[M]/\mathbb {M}_{\max }(G[M])$ and thus, the set $\mathcal {Q}$ can be computed $O (|X| + |E|)$ time. Note that $\mathcal {Q}$ has at most $|V(\mathscr {T}_G)|$ elements.

We consider now the runtime of one iteration of the for-loop starting in Line 6. To better distinguish between strong modules in $\mathbb {M}_{\textrm{str}}(G)$ and vertices in $(\mathscr {T}_G,\tau _G)$ (which are the elements in $\mathbb {M}_{\textrm{str}}(G)$) we use another notation. Let $H\in \mathcal {Q}$ as in Line 6. Then $H = G[M]/\mathbb {M}_{\max }(G[M])$ for some module $M\in \mathbb {M}_{\textrm{str}}(G)$ which is also vertex in $(\mathscr {T}_G,\tau _G)$ and we denote this vertex in the following by $v_H$. Moreover, put $n_H {:}{=} |{{\,\textrm{child}\,}}_{\mathscr {T}_G}(v_H)|$ and note that *H* contains exactly $n_H$ vertices, since $V(H)=\mathbb {M}_{\max }(G[M])={{\,\textrm{child}\,}}_{\mathscr {T}_G}(v_H)$. Let $m_H$ denote the number of edges in *H*. We first argue that determining if *H* is a near-cograph and, in the affirmative case, to find a vertex *z* resulting in the cograph $H-z$ can done in $O(n_H+m_H)$ time (Line 7–10). To achieve this goal, we can use the $O(n_H + m_H)$-time algorithm of Corneil et al. [48] that is designed to verify if a given graph is a cograph or not. Here, we use *H* as input for this algorithm. The algorithm is incremental and constructs the cotree of a subgraph $H[V']$ of *H* starting from a single vertex and then increasing $V'$ by one vertex at each step of the algorithm. Since *H* is not a cograph, at some point there is a set $V'$ and a chosen vertex *w* such that $H[V']$ is a cograph and $H[V'\cup \{w\}]$ contains an induced $P_4$. Based on these findings, Capelle et al. [74] showed that one can find an induced $P_4$ containing *w* in $O(\deg _H(w)) \subseteq O(n_H)$ time. Henceforth, we denote such an induced $P_4$ just by *P*. Now, we aim at finding a vertex *z* such that $H-z$ is a cograph. Due to Observation 4.9, *z* must be located on *P*, i.e., it is one of the four vertices in *P*. Thus, we check for all four vertices *z* in *P* again if $H-z$ is a cograph with the $O(n_H + m_H)$-time algorithm of Corneil et al. In summary, the tasks in Line 7–10 can be achieved in $O(n_H + m_H)$ time. The worst case for the runtime is of course the case that all graphs in $\mathcal {Q}$ are near-cographs. Thus, suppose that $H-z$ is a cograph. In this case, we can assume that the cotree $(T^H,t^H)$ of $H-z$ as required in Line 11 is already computed in the previous step. We now compute $(T^H_{{\nwarrow }z},t^H_{{\nwarrow }z})$ and add it to $\mathcal {P}(G)$ in Line 12. Note that *T* has precisely the vertices of *H* as its leaves and it is an easy task to verify that, therefore, $T^H_{{\nwarrow }z}$ can be constructed in $O(n_H)$ time. Keeping the labels of all $O(n_H)$ vertices in $T^H$ that are present in $T^H_{{\nwarrow }z}$ and adding labels to the $O(n_H)$ newly created vertices allows us to construct $t^H_{{\nwarrow }z}$ in $O(n_H)$ time. In summary, the time complexity to compute all tasks within a single iteration of the for-loop is in $O(n_H + m_H)$ for each $H\in \mathcal {Q}$.

We consider now the overall runtime of the for-loop. By construction, $|\mathcal {Q}| = |\mathbb {P}| \le |V(\mathscr {T}_G)|$. Hence, the the overall runtime of the for-loop can be expressed as $O(\sum _{v_H\in \mathbb {P}} (n_H + m_H)) = O(\sum _{v_H\in \mathbb {P}} n_H) + O(\sum _{v_H\in \mathbb {P}} m_H)$. As argued above, $n_H = |{{\,\textrm{child}\,}}_{\mathscr {T}_G}(v_H)|$ and hence, $O(\sum _{v_H\in \mathbb {P}} n_H )= O(\sum _{v_H\in \mathbb {P}} |{{\,\textrm{child}\,}}_{\mathscr {T}_G}(v_H)|) \subseteq O(|E(\mathscr {T}_G)|) = O(|V(\mathscr {T}_G)|) = O(|X|)$. In the following, we argue that $O(\sum _{v_H\in \mathbb {P}} m_H) \subseteq O(|E|)$. To this end, let $\{M_i,M_j\}$ be an edge in *H* for some $v_H\in \mathbb {P}$. This edge exists in *H* because there is an edge $\{x,y\}\in E$ with $x\in M_i$ and $y\in M_j$. Let us call such an edge $\{x,y\}$ a witness for the edge $\{M_i,M_j\}$. Hence, any edge in *H* is witnessed by an edge in *E*, i.e., $m_H\le |E|$ for all $v_H\in P$. Moreover, since any two distinct prime modules *M* and $M'$ of *G* are strong, it holds that (i) $M\cap M' = \emptyset$ or (ii) $M\subsetneq M'$ or (iii) $M'\subsetneq M$. In case (i) and (ii) we have $x\notin M'$ or $y\notin M'$. In case (ii), we have $\{x,y\}\subseteq M_k$ for some inclusion-maximal strong module $M_k\subsetneq M'$. In either case, the existence of any edge $\{M'_i,M'_j\}\in E(H')$ with $H' = G[M']/\mathbb {M}_{\max }(G[M'])$ is not witnessed by any edge that witnessed an edge in *H*. In other words, the set of edges that witness an edge in *H* and the set of edges that witness an edge in $H'$ are disjoint. Thus, $O(\sum _{v_H\in \mathbb {P}} m_H) \subseteq O(|E|)$. In summary, the overall runtime of the for-loop is in $O(|X|+|E|)$.

By construction, the network $(T^H_{{\nwarrow }z},t^H_{{\nwarrow }z})$ for every $H\in \mathcal {Q}$ contains the vertices of the MDT $\mathscr {T}_H$ of *H* whose leave set is of size $n_H = |{{\,\textrm{child}\,}}_{\mathscr {T}_G}(v_H)|$ and $O(n_H)$ additional vertices. It is now easy to verify that modifying $(\mathscr {T}_G,\tau _G)$ to ${{\,\textrm{pvr}\,}}(G,\mathcal {P}(G))$ can be done in $\sum _{v_H\in \mathbb {P}} n_H = \sum _{v_H\in \mathbb {P}} |{{\,\textrm{child}\,}}_{\mathscr {T}_G}(v_H)| \in O(|X|)$ time.

In summary, can be verified in $O(|X|+|E|)$ time if a given graph $G=(X,E)$ can be explained by a labeled level-1 network and, in the affirmative case, a 0/1-labeled level-1 network (*N*, *t*) that explains *G* can be constructed within the same time complexity. $\square$

### The substitution operator, perfect graphs, twin-width and related results

The latter results allow us to show that every Lev-1-Ex graph is obtained from Lev-1-Ex graph by “locally replacing” vertices by Lev-1-Ex graphs. To this end, we need the following

#### Definition 4.23

For two vertex-disjoint graphs *G* and *H* and a vertex $v\in V(G)$ we define the *substitution operation* as the graph defined by

1. Put  and
2. add the edge $\{u,w\}$ to $E(G')$ for all $\{u,v\}\in E(G)$ and for all $w\in V(H)$ which results in .

In this case, we say that *v* *is substituted by H* (in *G*).

In simple terms,  replaces the vertex *v* in *G* with the graph *H*, where the vertices in *H* are adjacent exactly to those vertices in *G* to which *v* was originally adjacent. The substitution operator is a standard tool in graph theory and has been heavily used, in particular, to prove the “perfect graph theorem” [75, 76] and to establish results related to primitive graphs or hereditary graph classes, see, e.g., [77–80]. In particular, Drgas-Burchardt [77] provides a characterization of those hereditary graph classes that are closed under the substitution operator. These results depend on some notions that differ significantly from our notation. Hence, instead of reusing these results, we provide a direct proof to ensure self-consistency.

#### Theorem 4.24

The class of Lev-1-Ex is closed under the substitution-operation . In particular, a graph *G* is Lev-1-Ex if and only if

(i) $G\simeq K_1$ or (ii)  with *H* and $H'$ being Lev-1-Ex and $|V(H')|>1$.

Furthermore, the disjoint union and the join of two Lev-1-Ex graph is a Lev-1-Ex graph.

#### Proof

Suppose that $G=(X,E)$ is Lev-1-Ex. If $|X|=1$ then $G\simeq K_1$ satisfies (i). Assume that $|X|>1$. In this case, let $K_1 = (\{v\},\emptyset )$. Thus,  and *G* satisfies (ii).

Suppose that $G = (X,E)$ satisfies (i) or (ii). If $G \simeq K_1$, then it is clearly Lev-1-Ex. Suppose that  for two Lev-1-Ex graphs *H* and $H'$, and a vertex $v\in V(H)$. If *G* contains no prime module, then Theorem 4.19 implies that *G* is Lev-1-Ex. Assume now that *M* is a prime module of *G* and put $W = V(H')$. By Lemma 4.3, *M* is strong. Note that all vertices in *W* have in *G*, by construction, the same neighbors and non-neighbors in $X\setminus W$, namely those of *v* in *H*. Hence, *W* is a module in *G*. Since *M* is strong, it does not overlap with *W*. Hence, there are three cases that we consider $M\cap W = \emptyset$, $W\subsetneq M$ or $M\subseteq W$. If $M\cap W = \emptyset$, then $G[M] = H[M]$ and thus, $\mathbb {M}_{\max }(G[M]) = \mathbb {M}_{\max }(H[M])$. Hence, $G[M]/\mathbb {M}_{\max }(G[M]) = H[M]/\mathbb {M}_{\max }(H[M])$. Since *H* is Lev-1-Ex, Proposition 4.14 implies that *H*[*M*] is Lev-1-Ex. In particular, *M* remains a prime module in *H*[*M*]. Thus, Theorem 4.19 implies that $H[M]/\mathbb {M}_{\max }(H[M]) = G[M]/\mathbb {M}_{\max }(G[M])$ is a near-cograph. If $M\subseteq W$, then $G[M] = H'[M]$ and we can reuse the latter arguments applied on $H'$ instead of *H* to conclude that $G[M]/\mathbb {M}_{\max }(G[M])$ is a near-cograph. If $W\subsetneq M$ then we consider $\mathbb {M}_{\max }(G[M]) = \{M_1,\dots ,M_k\}$. Since all $M_i\in \mathbb {M}_{\max }(G[M])$ are strong, they do not overlap with *W*. This and $W\subsetneq M$ implies that that $W\subseteq M_i$ for some $M_i\in \mathbb {M}_{\max }(G[M])$ which, in particular, implies that $H[M_j] = G[M_j]$ for all $j\in \{1,\dots k\}{\setminus } \{i\}$, and that the adjacencies between vertices in $M_j$ and $M_l$ remain unchanged for all $j,l \in \{1,\dots k\}\setminus \{i\}$. Moreover, by construction, $X = (V(H)\setminus \{v\} )\cup W$ and the adjacent vertices of $w \in W$ in $G[X\setminus W]$ are exactly the adjacent vertices of *v* in $H[V(H)\setminus \{v\}]$. The latter arguments imply that

$$\begin{aligned}\mathbb {M}_{\max }(H[M])=\{M_1,\ldots , M_{i-1},(M_i\setminus W)\cup \{v\},\ldots ,M_k\}\end{aligned}$$

and $G[M]/\mathbb {M}_{\max }(G[M]) \simeq H[M]/\mathbb {M}_{\max }(H[M])$ which, by Theorem 4.19, implies that $G[M]/\mathbb {M}_{\max }(G[M])$ is a near-cograph. Hence, for each of the cases $M\cap W = \emptyset$, $W\subsetneq M$ and $M\subseteq W$, the graph $G[M]/\mathbb {M}_{\max }(G[M])$ is a near-cograph and this holds for every prime module *M* of *G*. Theorem 4.19 implies that *G* is Lev-1-Ex. The latter arguments imply that the class of Lev-1-Ex is closed under the substitution-operation .

Finally, assume that *G* is the join or disjoint union of two Lev-1-Ex graphs $G_1$ and $G_2$. Suppose that *G* contains a prime module *M*. Hence, neither *G*[*M*] nor $\overline{G}[M]$ is connected. This directly implies that $M\subseteq V(G_i)$ for one $i\in \{1,2\}$. Therefore, $G[M] = G_i[M]$ and thus, $G[M]/\mathbb {M}_{\max }(G[M]) = G_i[M]/\mathbb {M}_{\max }(G_i[M])$. By Theorem 4.19, $G[M]/\mathbb {M}_{\max }(G[M])$ a near-cograph. As the latter arguments hold for all prime modules *M* of *G*, Theorem 4.19 implies that *G* is Lev-1-Ex. $\square$

We consider now “atomic expressions” $\textsf{T}$ of *G* that are based on the operations , , and . As already shown in Theorem 4.24, every Lev-1-Ex graph *G* can be written as a sequence of Lev-1-Ex graphs in which all graphs are concatenated by the -operator. We show now that every Lev-1-Ex graph can be written as a sequence of $K_1$’s and primitive near-cographs where the graphs are concatenated by the -, - and -operator. To be more precise:

#### Definition 4.25

For a graph class $\mathcal {G}$, we write $\underline{\mathcal {G}}$ to denote that one graph from $\mathcal {G}$ is taken. We denote by $\mathcal {N}$ the set of all primitive near-cographs and by $\mathcal {K}$ the set of all single-vertex graphs $K_1$. Moreover, when using , we always assume that *v* is a vertex in *H*. In addition, when using , , or , we always assume that *H* and $H'$ are vertex-disjoint.

An *atomic expression* ($\textsf{T}$) is an expression defined recursively by the following grammar:4An atomic expression $\textsf{T}$ is *valid for G* if, after substituting the elements $\underline{\mathcal {K}}$ and $\underline{\mathcal {N}}$ at each step with specified graphs from the respective classes $\mathcal {K}$ and $\mathcal {N}$, the result is a graph *H* that is isomorphic to *G*. In this case, we also say that $\textsf{T}$ is a *valid* atomic expression for *G*. Moreover, we write $\textsf{T}(G)$ for one of the valid atomic expression for *G*, if it exists.

We provide now a couple of examples for graphs that have a valid atomic expression. The simplest one is provided by the single vertex graph $K_1$ for which both $\underline{\mathcal {K}}$ and  are valid atomic expressions, illustrating that there can be multiple valid atomic expressions for the same graph.

Now, consider the graph *G* as in Fig. 5 and let $P = G[\{a,b,c,d\}] \in \mathcal {N}$. Moreover, put $K^v {:}{=} (\{v\},\emptyset )$ to specify the vertex set of a $K_1\in \mathcal {K}$. In this example,
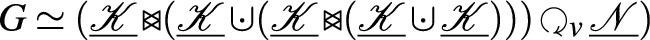


where the specified graph $\underline{\mathcal {N}}$ is an induced $P_4$ and, in particular,
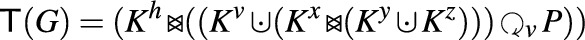


is a valid atomic expression of *G*. In other words, *G* is composed of $K_1$s and primitive near-cographs linked by ,  and . Another valid atomic expressions for *G* is .

The graph *G* in Fig. 3 is just the disjoint union of primitive near-cographs, namely $P_4$s and we obtain the valid atomic expression 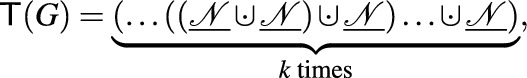 where each specified graph $\underline{\mathcal {N}}$ is an induced $P_4$.

Clearly, every graph can be written as  with $K = (\{v\}, \emptyset )$. However, not all graphs admit a valid atomic expression. For example, an induced cycle $C_5$ on five vertices is primitive but not Lev-1-Ex (cf. Lemma 4.28). Hence, $C_5 \notin \mathcal {K} \cup \mathcal {N}$. In particular, it is easy to verify that an induced $C_5$ has no valid atomic expression. In contrast, an induced cycle $C_4$ has a valid atomic expression, namely .

#### Theorem 4.26

A graph *G* is Lev-1-Ex if and only if there exists a valid atomic expression $\textsf{T}(G)$ for *G*.

#### Proof

We prove the *only-if* direction by induction on the number of vertices in $G = (X,E)$. If $|X|=1$, then $\textsf{T}(G) = \underline{\mathcal {K}}$ is a valid atomic expression of *G*. Suppose now that all Lev-1-Ex graphs *G* with $|X|\le n$ vertices have a valid atomic expression $\textsf{T}(G)$ for some $n\ge 1$. Let $G = (X,E)$ be a graph with $|X| = n+1 > 1$ vertices and consider the root $\rho$ of the MDT $(\mathscr {T},\tau )$ of *G*. Since $|X|>n$, it follows that for the set of children $\mathbb {M}_{\max }(G) = \{M_1,\dots ,M_k\}$ of $\rho$ it holds that $k>1$ and $|M_i|\le n$. By Proposition 4.14, $G_i{:}{=} G[M_i]$ is Lev-1-Ex for all $i\in \{1,\dots ,k\}$. By induction hypothesis, $G_i$ has a valid atomic expression $\textsf{T}(G_i)$ for all $i\in \{1,\dots ,k\}$.

We consider now the three possible labels of $\rho$. If $\tau (\rho ) = 0$ then  (cf. Observation 4.1) and it follows that  is a valid expression for *G*. If $\tau (\rho ) = 1$ then  (cf. Observation 4.1) and it follows that  is a valid expression for *G*. Assume now that $\tau (\rho ) = \textrm{prime}$. Since *G* is Lev-1-Ex, Lemma 4.3 implies that $H{:}{=} G[X]/\mathbb {M}_{\max }(G[X])= G/\mathbb {M}_{\max }(G)$ is a primitive Lev-1-Ex graph and, by Theorem 4.12, a primitive near-cograph. Consider first the possibility that $|V(H)|=n+1$. In this case, $|V(H)|=|\mathbb {M}_{\max }(G)|=|X|$ which enforces $|M_i|=1$ for each $M_i\in \mathbb {M}_{\max }(G)$ and, therefore, $G\simeq H$. In particular,  is a valid atomic expression for *G*, obtained by specifying $\underline{\mathcal {N}}$ to be the primitive near-cograph *H* and $\underline{\mathcal {K}}$ to be the $K_1$ defined as $(\{v\},\emptyset )$. Henceforth assume that $|V(H)|\le n$. By induction hypothesis, there is a valid atomic expression $\textsf{T}(H)$ of *H*. Moreover *G* is obtained from *H* by substituting each vertex $M_1,\dots ,M_k$ by $G_1,\dots ,G_k$, respectively. In other words, . Hence, 

is a valid atomic expression of *G*.

We now proceed with the *if* direction by induction on the number of terminal symbols in $\textsf{T}(G)$, that is, on the number of occurrences of $\underline{\mathcal {K}}$ and $\underline{\mathcal {N}}$ in $\textsf{T}(G)$. In what follows, we make frequent use of the fact that, for every atomic expression $\textsf{T}$, there is a graph *G* with $\textsf{T}(G) = \textsf{T}$. If $\textsf{T}(G)=\underline{\mathcal {K}}$ then *G* is isomorphic to a $K_1$ and thus clearly Lev-1-Ex. If $\textsf{T}(G)=\underline{\mathcal {N}}$, then *G* is a primitive near-cograph and by Theorem 4.12 also Lev-1-Ex. The latter two arguments comprise the base case of one terminal symbol appearing in $\textsf{T}(G)$. Assume that, for some $n\ge 1$ and for every atomic expression $\textsf{T}(G)$ with at most *n* terminal symbols, the graph *G* is a Lev-1-Ex graph. Now let *G* be a graph with a valid atomic expression $\textsf{T}(G)$ that contains $n+1$ terminal symbols. By definition of atomic expressions and since $n+1>1$, we have ,  or  for some atomic expressions $\textsf{T}'$ and $\textsf{T}''$. Let $G'$ and $G''$ be two graphs for which $\textsf{T}(G') = \textsf{T}'$ and $\textsf{T}(G'') = \textsf{T}''$. Since $\textsf{T}'$ and $\textsf{T}''$ have at least one terminal symbol each, neither have more than *n* terminal symbols and we can apply the induction hypothesis to conclude that $G'$ and $G''$ are Lev-1-Ex graphs. Hence it follows that ,  or . In all three cases, Theorem 4.24 implies that *G* is Lev-1-Ex. $\square$

We note in passing that the disjoint union and join of two graphs *G* and $G'$ can alternatively be written as  and  where $K_2 = (\{u,v\}, (\{u,v\}))$ is the graph with two vertices *u* and *v* connected by an edge. Let us denote with $\mathcal {K}^+$ the set of all graphs isomorphic to $K_1$, $K_2$ and $\overline{K_2}$. Using the notation introduced in Eq. 4, we can thus write both  and  as . This, together with the derivation “$\textsf{T}\rightarrow \textsf{A} \rightarrow \underline{\mathcal {K}^+}$” and Theorem 4.26, leads to the following result, which essentially mirrors [77, Cor. 1] for Lev-1-Ex graphs.

#### Corollary 4.27

A graph *G* is Lev-1-Ex if and only if there exists a valid expression for *G* in the grammar recursively defined by 
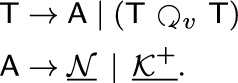


We close this section by showing the relationship between Lev-1-Ex graphs and other graph classes, as well as their connection to the “twin-width”. A graph *G* is *perfect* if the chromatic number of every induced subgraph equals the size of the largest clique in that subgraph. It is well-known that perfect graphs are closed under the substitution operation (cf. [75, Thm 1]). By Theorem 4.24, Lev-1-Ex graphs also possess this property. Hence, it is natural to ask whether Lev-1-Ex graphs are perfect. An affirmative answer to this question is provided in Theorem 4.29. To establish this result, we first define a *hole* as an induced cycle $C_n$ on $n \ge 5$ vertices. The complement of a hole is called an *anti-hole*.

#### Lemma 4.28

Lev-1-Ex graphs are hole-free and anti-hole-free.

#### Proof

By contraposition, suppose that *G* contains an induced $C_n$ on $n\ge 5$ vertices, i.e., $G[W]\simeq C_n$ for some $W\subseteq V(G)$. One easily verifies that *G*[*W*] is primitive and not a near-cograph. By Lemma 4.12, *G*[*W*] is not Lev-1-Ex and, by Proposition 4.14, *G* is not Lev-1-Ex. Hence, Lev-1-Ex graphs are hole-free. Assume now that *G* is Lev-1-Ex. By Observation 3.2, ${\overline{G}}$ is Lev-1-Ex. By the latter arguments, ${\overline{G}}$ must be hole-free which implies that *G* is anti-hole-free. $\square$

A graph is *weakly-chordal* if and only if it does not contain holes or anti-holes [81]. As shown by Hayward in [81], weakly-chordal graphs are perfect. The latter together with Lemma 4.28 implies

#### Theorem 4.29

Lev-1-Ex graphs are weakly-chordal and thus, perfect.

Bonnet et al. recently introduced a novel parameter called “twin-width (${{\,\textrm{tww}\,}}$)” as a non-negative integer measuring a graphs distance to being a cograph [43] that is based one the following characterization of cographs: A graph is a cograph if it contains two vertices with the same neighborhood (called twins), identify them, and iterate this process until one ends in a $K_1$ [42, 43]. This makes cographs the unique class of graphs having twin-width 0. We omit the (lengthy) formal definition of twin-width here and instead refer the reader to [43].

#### Proposition 4.30

If *G* is a Lev-1-Ex graph, then ${{\,\textrm{tww}\,}}(G)\le 2$. There exists Lev-1-Ex graphs with twin-width equal to 2.

#### Proof

We first remark that [43, Thm 4.1] states that ${{\,\textrm{tww}\,}}(G)\le 2({{\,\textrm{tww}\,}}(G-v)+1)$ for all graphs *G* and all vertices *v* of *G*. Since every near-cograph *H* has, by definition, a vertex *v* such that $H-v$ is a cograph and thus such that ${{\,\textrm{tww}\,}}(H-v)=0$, every near-cograph *H* satisfies ${{\,\textrm{tww}\,}}(H)\le 2$. Furthermore note that, by [82, Thm. 3.1], we have

$$\begin{aligned} {{\,\textrm{tww}\,}}(G) = \max \{&{{\,\textrm{tww}\,}}(G[M]/\mathbb {M}_{\max }(G[M]))\,: \\  &\,M \text { is a prime module of }G\}, \end{aligned}$$

for all graphs *G* and thus Theorem 4.19 implies that the twin-width of any Lev-1-Ex graph *G* equals the maximum twin-width of a set of near-cographs. As previously argued, each such near-cograph has twin-width at most 2, thus so does *G*. In summary, ${{\,\textrm{tww}\,}}(G)\le 2$ for all Lev-1-Ex graphs *G*.

We show now that there are Lev-1-Ex graphs for which this bound is tight. To this end, consider the graph $G=(V,E)$ where $V=\{x,v_1,v_2,v_3,u_1,u_2,u_3\}$ and whose edges are $\{x,v_i\}$ and $\{v_i,u_i\}$ for $i=1,2,3$. Since $G-x$ is a cograph, *G* is a near-cograph and Corollary 4.11 implies that *G* is Lev-1-Ex. We show now that ${{\,\textrm{tww}\,}}(G)=2$. The graph *G* contains an *asteroidal triple* $(u_1,u_2,u_3)$, i.e., $u_1,u_2,u_3$ are pairwise non-adjacent vertices and $u_i$ and $u_j$ are connected by a path in *G* which does not contain vertices that are adjacent to the vertex $u_k$, $\{i,j,k\} = \{1,2,3\}$ [83]. In particular, Theorem 4 in [83] implies that *G* is not a so-called co-comparability graph. Therefore, [84] (c.f. [85, Thm. 4.7.1]) implies that *G* is not a so-called permutation graph. Now Theorem 8 in [86] implies that ${{\,\textrm{tww}\,}}(G)\ge 2$. Since *G* is Lev-1-Ex the previous arguments imply that ${{\,\textrm{tww}\,}}(G)=2$. $\square$

## Summary and outlook

In this contribution, we have established a general framework for evaluating the biological feasibility of orthology graphs. In particular, we examined whether a network-based evolutionary scenario exists that allows for speciation events, represented by a 0/1-labeling of its inner vertices, that give rise to the orthologs encoded in the orthology graph. We demonstrated that for every orthology graph *G*, there exists a network *N* with a 0/1-labeling that explains *G*, where *N* has no more hybrid vertices than *G* has vertices (cf. Theorem 3.10). In this sense, *every* inferred set of orthologous gene pairs can, in principle, be represented within some network-based evolutionary scenario. However, such a scenario may be excessively complex and biologically implausible. It is therefore natural to impose constraints on the complexity of the permissible networks. To this end, we focused on level-1 networks, which, in an intuitive sense, are close to being tree-like while still allowing for occasional non-tree-like evolutionary events. We provided several characterizations of Lev-1-Ex graphs, i.e., orthology graphs that can be explained by level-1 networks. In this context, modular decomposition proved to be a useful tool for exploring the non-tree-like behavior of networks explaining a graph *G*, as such behavior is determined by the structural properties within the prime modules of *G*. In particular, *G* is Lev-1-Ex if and only if each primitive subgraph is a near-cograph. Clearly, if an orthology graph *G* lacks this structure, it would be necessary to determine the appropriate edits (removal or addition of edges) required to transform it into a Lev-1-Ex graph. The computational complexity of the underlying editing problem remain an interesting open question for future research.

We propose that modular decomposition can serve as a toolbox for further generalizations, particularly for characterizing orthology graphs that can be explained by network types beyond level-1. Specifically, if the primitive subgraphs of an orthology graph *G* can be explained by a given network type, then it is plausible that the entire graph *G* can also be explained by the same type of network. A reasonable approach to proving this assumption for certain network types is to employ prime-vertex replacement networks (cf. Definition 4.16). These networks are used to replace prime vertices in the modular decomposition tree of *G* with specified networks that explain the underlying primitive subgraphs. However, even the natural generalization to level-2 networks appears to be non-trivial. As illustrated in Fig. 6, additional hybridization events introduce significantly greater structural complexity to the underlying orthology graph. Nevertheless, understanding these generalizations could provide deeper insights into how hybridization affects the representation of orthology relationships.

Another important aspect concerns the relationship between orthologs and best matches. This topic has received considerable attention in recent years under the assumption of tree-like evolution [87–89]. An orthology graph that can be explained by a tree must always be a subgraph of the (reciprocal) best match graph underlying this tree [89]. Moreover, in [90] it was shown that, given the correct best match graph, it is possible to optimally correct estimates of orthologs in polynomial time to obtain a tree-explainable orthology graph. A natural question arises: how do best matches relate to orthology in the context of networks? Addressing this question requires not only a deeper understanding of orthologs but also a more detailed investigation of best matches and their relationship to orthology in network-based evolutionary scenarios.

Finally, our results open new avenues for further research in graph theory, particularly in understanding the broader mathematical properties of orthology graphs and their relationships to other graph classes or graph parameters. Investigating these connections could lead to novel insights that extend beyond the scope of orthology inference.

## Data Availability

No datasets were generated or analysed during the current study.
